# Triggering the expression of a silent gene cluster from genetically intractable bacteria results in scleric acid discovery†
†Electronic supplementary information (ESI) available: Supplementary methods and results; Tables S1–S6; Fig. S1–S22. See DOI: 10.1039/c8sc03814g


**DOI:** 10.1039/c8sc03814g

**Published:** 2018-10-19

**Authors:** Fabrizio Alberti, Daniel J. Leng, Ina Wilkening, Lijiang Song, Manuela Tosin, Christophe Corre

**Affiliations:** a Warwick Integrative Synthetic Biology Centre and School of Life Sciences , University of Warwick , Coventry , CV4 7AL , UK . Email: F.Alberti@warwick.ac.uk ; Email: C.Corre@warwick.ac.uk; b Department of Chemistry , University of Warwick , Coventry , CV4 7AL , UK

## Abstract

In this study, we report the rapid characterisation of a novel microbial natural product resulting from the rational derepression of a silent gene cluster. A conserved set of five regulatory genes was used as a query to search genomic databases and identify atypical biosynthetic gene clusters (BGCs). A 20-kb BGC from the genetically intractable *Streptomyces sclerotialus* bacterial strain was captured using yeast-based homologous recombination and introduced into validated heterologous hosts. CRISPR/Cas9-mediated genome editing was then employed to rationally inactivate the key transcriptional repressor and trigger production of an unprecedented class of hybrid natural products exemplified by (2-(benzoyloxy)acetyl)-l-proline, named scleric acid. Subsequent rounds of CRISPR/Cas9-mediated gene deletions afforded a selection of biosynthetic gene mutant strains which led to a plausible biosynthetic pathway for scleric acid assembly. Synthetic standards of scleric acid and a key biosynthetic intermediate were also prepared to confirm the chemical structures we proposed. The assembly of scleric acid involves two unique condensation reactions catalysed by a single NRPS module and an ATP-grasp enzyme that link a proline and a benzoyl residue to each end of a rare hydroxyethyl-ACP intermediate, respectively. Scleric acid was shown to exhibit moderate inhibition activity against *Mycobacterium tuberculosis*, as well as inhibition of the cancer-associated metabolic enzyme nicotinamide *N*-methyltransferase (NNMT).

## Abstract 2

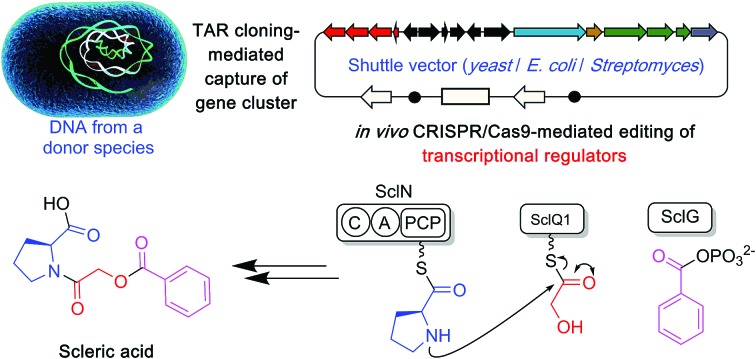
The characterisation of scleric acid, a new natural product from a silent and cryptic gene cluster from genetically intractable bacteria, and its biosynthesis are reported.

## Introduction

Actinomycete bacteria have been the foremost producers of antibiotics since the mid-1940s. In the last decade, high-throughput DNA sequencing technologies and novel bioinformatics tools have highlighted an immense number of uncharacterised biosynthetic gene clusters (BGCs) predicted to direct the assembly of bioactive natural products.^1^ The presence of BGCs has not only been revealed in actinomycete genomes but also in those of human commensal and pathogenic bacteria (*i.e. Staphylococcus lugdunensis*, *Burkholderia cepacia* complex), as well as in the genomes of unculturable bacteria and in metagenomic libraries.^2^–^7^

Despite the conspicuous number of specialised metabolites isolated from actinomycetes, only a small fraction of the natural products ‘encrypted’ at the DNA level has been exploited to date. Experimental characterisation of the biosynthetic product of a BGC is often laborious and time-consuming particularly due to the uniqueness of every microorganism. Protocols for introducing DNA into bacterial cells are species-dependent and often ineffective. Their optimisation can take years but many culturable micro-organisms remain genetically intractable. This prevents the exploitation of BGCs using many of the previously reported strategies.^1^ In addition, the biosynthesis of specialised metabolites is often tightly controlled at the transcriptional level. Cluster-associated transcriptional regulators that belong to the TetR-family of transcriptional repressors are particularly numerous.^8^ Deletions of cluster-specific TetR-like transcriptional repressors have been shown to trigger overproduction of the corresponding specialised metabolites, as previously reported for the antibiotics methylenomycin and coelimycin in *Streptomyces coelicolor* A3(2) and for the urea-containing gaburedins in *Streptomyces venezuelae*.^9^–^12^

Genetic manipulation of *Streptomyces* genomes has classically been accomplished using established but often laborious protocols optimised for specific bacterial strains.^13^ In recent years however, targeted genome editing has been revolutionised by the advent of clustered regularly interspaced short palindromic repeats (CRISPR)/CRISPR-associated (Cas) systems, which allow generation of clean genomic deletions/insertions.^14^ Toolkits for editing streptomycete genomes have been developed,^15^–^17^ enabling researchers to overcome the issues associated with classical methods of gene disruption, in particular when multiple mutation events are desirable.^18^ The number of available selectable markers and issues with potential restoration of the wild-type configuration due to occurrence of single-crossover events were notable limitations.

**Fig. 1:**
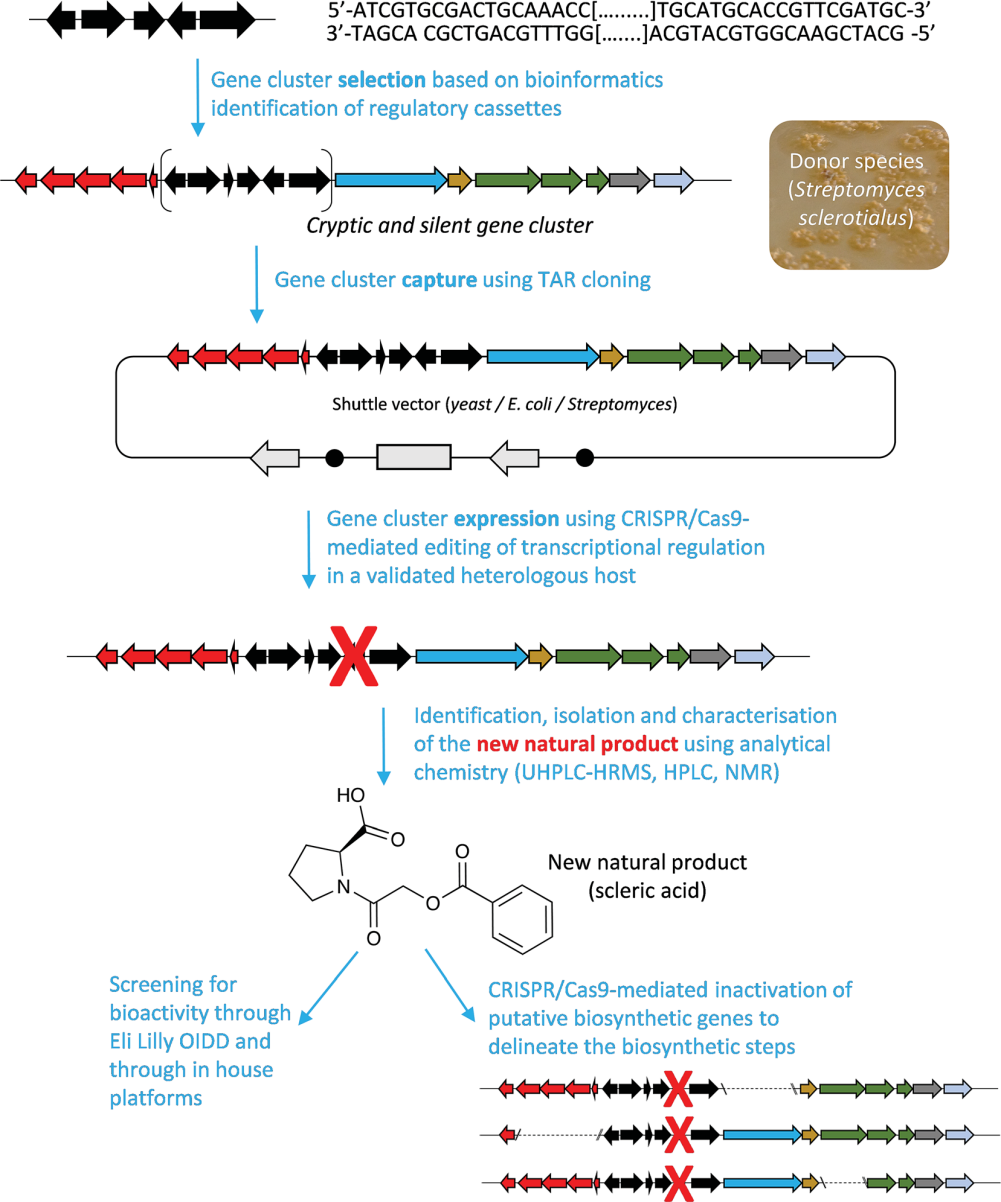
Overview of the approach used in this study to characterise scleric acid, a novel natural product from a cryptic and silent gene cluster.

Here, we report a genome mining strategy based on the identification of a conserved regulatory cassette for selecting and characterising BGCs. A specific BGC was first captured and transferred into a validated *Streptomyces* heterologous host where CRISPR/Cas9-mediated genome editing was employed to rationally derepress the expression of silent biosynthetic genes (Fig. 1). This approach was applied for the identification, isolation and structural elucidation of a novel structural class of natural products from a silent and cryptic gene cluster found in the soil-dwelling species *Streptomyces sclerotialus* NRRL ISP-5269, a species of filamentous bacteria first isolated in Poona (India).^19^

## Results and discussion

### Identification of the *scl* gene cluster in *S. sclerotialus* NRRL ISP-5269

The prioritisation of the gene cluster under study was guided by the presence of a specific set of five regulatory genes that we had previously characterised in *S. coelicolor* A3(2),^9^ and subsequently exploited to trigger the expression of a silent and cryptic biosynthetic gene cluster in *Streptomyces venezuelae*.^12^ In *S. coelicolor* A3(2), these five genes are responsible for regulation of methylenomycin biosynthesis: *mmyR* and *mmfR* both code for TetR-like transcriptional repressors; *mmfLHP* are responsible for the biosynthesis of signalling molecules, known as methylenomycin furans (MMFs) that trigger production of the methylenomycin antibiotics.^9^ A mathematical model that explains in detail the methylenomycin regulatory system involving these five genes has been developed and matched with experimental data.^20^ In previous studies we have exploited this regulatory cassette and shown that inactivation of the *mmyR*-like transcriptional repressors is an effective approach for derepressing silent gene clusters in actinomycetes.^10^,^12^

In order to find gene clusters that contained regulatory cassettes homologous to the one found in the methylenomycin cluster and to assess how widespread these are, we performed searches with Multigene BLAST^21^ using as a query the DNA sequences of *mmyR*, *mmfR* and *mmfLHP*, as well as with ClusterTools,^22^ using as a query the protein sequences of MmyR, MmfR and MmfLHP. Fourteen actinomycete genomes were found that contained orthologues of all five genes coding for MmyR, MmfR and MmfLHP within a 50-kb region (ESI Table S4†). Remarkably, a total of 98 actinomycete genomes were found that contained orthologues of at least *mmyR*, *mmfR* and *mmfL* within a 50-kb region. We have previously shown that the butenolide synthase MmfL alone is sufficient to give production of functional MMF signalling molecules in *S. coelicolor* A3(2).^9^ Additionally, a functional regulatory system that controls biosynthesis of coelimycin antibiotics in *S. coelicolor* A3(2) has been characterised that includes the butenolide synthase ScbA and the two TetR-like transcriptional regulators ScbR and ScbR2,^11^ which are orthologues of MmfL, MmfR and MmyR respectively. Hence the regulatory systems that include orthologues of *mmyR*, *mmfR* and *mmfL* are also putatively functional, and the biosynthetic products they regulate expression of could also be studied *via* manipulation of these regulatory cassettes.

**Fig. 2:**
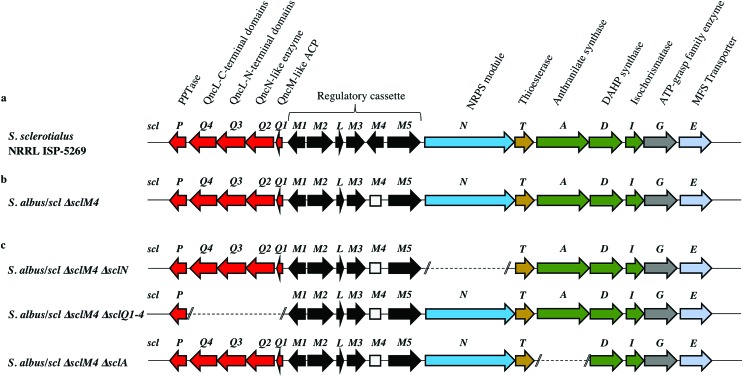
Genetic organisation of the *scl* gene cluster from *S. sclerotialus* NRRL ISP-5269 characterised in this study and schematic representation of mutants generated. (a) Gene cluster (19 782 bp) sequenced from *S. sclerotialus* NRRL ISP-5269. (b) Configuration of the *scl* gene cluster after CRISPR/Cas9-mediated targeted gene deletion within the heterologous host *S. albus*. The 20-bp out of frame deletion of *sclM4* generated a truncated gene, represented here as a rectangular shape for the derived gene sequence. (c) Configuration of the *scl* cluster within *S. albus* double mutants; deletion of genes *sclN*, *sclQ1-4* and *sclA* were generated starting from strain *S. albus*/*scl* Δ*sclM4*, and are represented here as dotted lines.

Among the hits generated, the gene cluster from *S. sclerotialus* NRRL ISP-5269, named hereafter *scl* cluster, was chosen for further study; the nucleotide sequence containing the *scl* cluster was available from the GenBank accession number JOBC01000043.1. In addition to homologues of the five genes used as query, the genetic organisation of the *scl* gene cluster included two adjacent and divergent operons of biosynthetic genes (Fig. 2a). A combination of AntiSMASH^23^ and manual BLASTp^24^ analyses indicated the putative borders of the *scl* cluster (Fig. 2a and Table 1). The predictive power of these modern bioinformatics tools often permits to deduce the chemical structure(s) of cryptic gene cluster products, particularly when modular systems such as type I modular polyketide synthases (PKS) or non-ribosomal peptide synthetases (NRPS) direct the biosynthesis.^25^ However, the originality of the *scl* cluster prevented such predictions and we worked on the assumption that a lack of bioinformatics prediction was more likely to result in a more structurally diverse and therefore truly novel natural product. The cluster spanned a region of 19 782 bp, and comprised 18 putative genes: 11 biosynthetic genes, 6 genes for regulation and 1 gene coding for a membrane transporter (Fig. 2a and Table 1).

**Table 1:** Proposed function of the biosynthetic, regulatory and resistance genes from the *scl* gene cluster

Protein (number of aa) GenBank	Homologue (% identity/% similarity) organism GenBank	Putative function	Proposed role
SclP (213) WP_037773640.1	4-Phosphopantetheinyl transferase (54/61) *Streptomyces pristinaespiralis* WP_078951206.1	PPTase	Biosynthesis of the glycolic acid unit
SclQ4 (386) WP_030624999.1	QncL (37/49) *Streptomyces melanovinaceus* AFJ11255.1 (ref. 34)	Lipoyl attachment domain, acyltransferase catalytic domain	
SclQ3 (338) WP_030625001.1	QncL (51/68) *S. melanovinaceus* AFJ11255.1 (ref. 34)	Pyrimidine binding domain, transketolase C-terminal domain	
SclQ2 (305) WP_078889003.1	QncN (61/73) *S. melanovinaceus* AFJ11257.1 (ref. 34)	ThDP binding domain	
SclQ1 (76) WP_030625009.1	QncM (32/68) *S. melanovinaceus* AFJ11256.1 (ref. 34)	Acyl carrier protein (ACP)	

SclM1 (200) WP_078889004.1	MmfR (56/73) *Streptomyces coelicolor* A3(2) WP_011039544.1 (ref. 9 and 10)	TetR-family transcriptional repressor	Regulation of the expression of scleric acid biosynthetic genes
SclM2 (336) WP_078889005.1	MmfL (35/47) *S. coelicolor* A3(2) WP_011039545.1 (ref. 9 and 10)	Signaling molecule biosynthesis (butenolide synthase)	
SclL (106) WP_051872433.1	LysR family transcriptional regulator (61/72) *S. venezuelae* WP_015035381.1	LysR transcriptional regulator	
SclM3 (228) WP_078889008.1	MmfP (46/56) *S. coelicolor* A3(2) WP_011039547.1 (ref. 9 and 10)	Signaling molecule biosynthesis (hydrolase)	
SclM4 (196) WP_051872434.1	MmyR (41/56) *S. coelicolor* A3(2) WP_011039548.1 (ref. 9 and 10)	TetR-family transcriptional repressor	
SclM5 (378) WP_051872435.1	MmfH (49/59) *S. coelicolor* A3(2) WP_011039546.1 (ref. 9 and 10)	Signaling molecule biosynthesis (oxidoreductase)	

SclN (1083) WP_078889006.1	PuwA (32/48) *Cylindrospermum alatosporum* CCALA 988 AIW82277.1 (ref. 32)	NRPS [C-A-PCP]	Activation of l-proline and condensation with glycolic acid

SclT (243) WP_030625041.1	Thioesterase (45/56) *Streptomyces sviceus* WP_007379259.1	Thioesterase (TE)	Hydrolytic release of scleric acid from a carrier protein

SclA (656) WP_051872438.1	PauY18 (56/67) *Streptomyces* sp. YN86 AIE54238.1 (ref. 33)	Anthranilate synthase	Biosynthesis of the benzoic acid unit
SclD (405) WP_030625030.1	PauY21 (55/65) *Streptomyces* sp. YN86 AIE54241.1 (ref. 33)	DAHP synthase	
SclI (220) WP_030625033.1	PauY19 (57/71) *Streptomyces* sp. YN86 AIE54239.1 (ref. 33)	Isochorismatase	

SclG (439) WP_030625035.1	ATP-grasp domain-containing protein (49/62) *Streptomyces* sp. NRRL B-5680 WP_051746523.1	ATP-grasp family enzyme	Condensation reaction of the proline unit with benzoic acid

SclE (435)	Major Facilitator Superfamily transporter (53/65) *Amycolatopsis vancoresmycina* WP_003090471.1	MFS transporter	Export of scleric acid outside the cell

### Capture of the *scl* gene cluster and introduction into heterologous hosts

In order to characterise the product of the *scl* cluster, we first set out to examine the genetic tractability of *S. sclerotialus* NRRL ISP-5269. To do so, we attempted introduction of plasmid pCRISPomyces-2 (ref. 15) for CRISPR/Cas9-based genome engineering *via* intergeneric-conjugation of mycelia with *E. coli* ET12567/pUZ8002 or protoplast transformation but these processes were found ineffective.^13^ The preparation of *S. sclerotialus* NRRL ISP-5269 spores was also attempted but despite screening various culture media in the aim to induce sporulation, this strain showed little aerial growth. We therefore set out to capture and heterologously express the *scl* gene cluster, with the aim of characterising its biosynthetic product(s) in a well-characterised and genetically amenable host. A 33-kb region of genomic DNA that included the 19.8-kb *scl* BGC was captured from the purified genomic DNA of *S. sclerotialus* ISP-5269 *via* transformation-associated recombination (TAR) cloning (ESI Fig. S1†).^26^ A pCAP03-derived plasmid was first assembled to specifically recombine in yeast with both extremities of the 33-kb target DNA fragment.^27^ The pCAP03-*scl* construct was introduced and stably integrated into the genome of *Streptomyces albus* J1074 and *S. coelicolor* M1152 *via* intergeneric tri-parental conjugation with *E. coli* ET12567/pCAP03-*scl* and *E. coli* ET12567/pUB307. These two strains were chosen as they are well characterised chassis for heterologous expression of actinomycete gene clusters.^28^,^29^ In parallel, the negative control strains *S. albus*/pCAP03 and *S. coelicolor* M1152/pCAP03 were generated using the “empty” pCAP03 plasmid. The four *Streptomyces* strains were grown on supplemented minimal agar medium for 5 days, and the acidified agar medium was extracted with ethyl acetate. Their metabolic profiles were analysed by ultra-high-pressure liquid chromatography-high resolution mass spectrometry (UHPLC-HRMS). Comparison of the MS chromatograms failed to reveal any new compounds in the heterologous hosts where the *scl* BGC had been integrated.

### Derepression of the *scl* gene cluster and characterisation of scleric acid

**Fig. 3:**
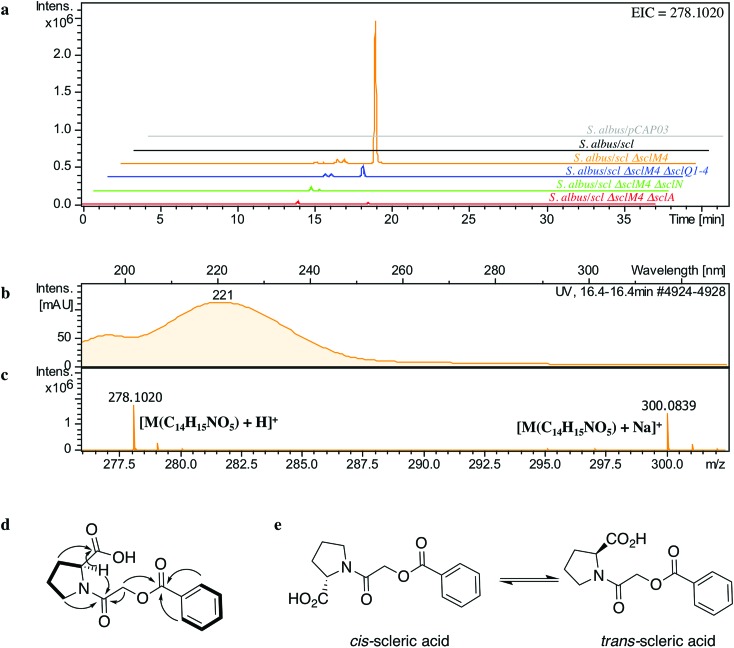
Identification and characterisation of scleric acid. (a) UHPLC-HRMS detection of metabolites produced in *S. albus*/*pCAP03* (grey trace), *S. albus*/*scl* (black), *S. albus*/*scl* Δ*sclM4* (orange), *S. albus*/*scl* Δ*sclM4* Δ*sclQ1-4* (blue), *S. albus*/*scl* Δ*sclM4* Δ*sclN* (green) and *S. albus*/*scl* Δ*sclM4* Δ*sclA* (red). Extracted ion chromatograms in positive mode for *m*/*z* = 278.1020 are shown, highlighting accumulation of a new metabolite (scleric acid) at retention time 16.4 minutes in *S. albus*/*scl* Δ*sclM4* (orange trace). (b) UV chromatogram of scleric acid. (c) High-resolution mass spectrum in positive mode of scleric acid. (d) Selected correlations observed in the COSY (bold lines) and HMBC (arrows) NMR spectra of scleric acid. (e) Chemical structure of scleric acid that can adopt two main rotamer conformations.

The lack of accumulation of novel metabolites in the *scl*-containing strains was likely to be due to the transcriptional repression activity of the TetR-like regulators encoded in the *scl* cluster. We had previously shown that genetic inactivation of *mmyR*-like genes in *S. coelicolor* A3(2) and in *S. venezuelae* would specifically derepress the expression of adjacent BGCs.^10^,^12^ We therefore decided to genetically inactivate the *mmyR* homologous gene, *sclM4* using the CRISPR/Cas9-based plasmid pCRISPomyces-2 (pCm2).^15^ For this purpose, we assembled the plasmid pCm2-*sclM4* and attempted intergeneric-conjugation of *S. albus*/*scl* and *S. coelicolor* M1152/*scl* with *E. coli* ET12567/pUZ8002/pCm2-*sclM4*. Ex-conjugants for strain *S. albus*/*scl* Δ*sclM4* (see Fig. 2b for a representation of the genotype) were readily obtained, however no ex-conjugants could be obtained when attempting insertion of plasmid pCm2-*sclM4* into *S. coelicolor* M1152/*scl*. The desired 20-bp out-of-frame deletion of *sclM4* was confirmed by sequencing of a PCR product using *S. albus*/*scl* Δ*sclM4* genome as a template (ESI Fig. S2†). Metabolites produced by this strain were compared by UHPLC-HRMS to those of *S. albus*/*scl* and *S. albus*/*pCAP03* grown under the same conditions as those described earlier. *S. albus*/*scl* Δ*sclM4* showed accumulation of a major metabolite (Fig. 3a–c) with a retention time of 16.4 minutes on C_18_ reverse phase HPLC column and an *m*/*z* value of 278.1020 [M(C_14_H_15_NO_5_) + H]^+^ (calculated *m*/*z* of 278.1023). This compound was purified using a combination of flash chromatography on C_18_-silica column and HPLC. Its structure was then elucidated by a combination of 1D- and 2D-NMR spectroscopy experiments (ESI Fig. S5–S9†). The novel compound was characterised as being (2-(benzoyloxy)acetyl)-l-proline and named scleric acid (Fig. 3d).

In order to establish the stereochemistry of the proline residue, scleric acid was hydrolysed and derivatised with Marfey's reagent.^30^l- and d-proline were also derivatised using the same procedure and used as standards for HPLC comparison. Approximately 95% of the proline residue of scleric acid purified from *S. albus*/*scl* Δ*sclM4* was found to correspond to l-proline (ESI Fig. S10†). To confirm the proposed structure of scleric acid, an authentic standard was synthesised (see ESI Fig. S20† for a schematic representation of the synthetic route). A structural analogue, 2-((benzoyl-l-prolyl)oxy)acetic acid, possibly consistent with the initial NMR data obtained, was also synthesised (see ESI Fig. S21† for a schematic representation of the synthetic route and ESI Fig. S11–S15† for NMR spectra). LC-MS analyses and NMR data of both of these compounds unequivocally confirmed the proposed structure for scleric acid (Fig. 3); the analogue revealed different NMR spectra and its physico-chemical properties resulted in a different retention time on LC-MS. Moreover, two sets of NMR signals were observed for the purified natural product as well as for the synthetic standard and revealed that scleric acid existed as two different rotamers, *trans*- and *cis*-scleric acid (Fig. 3e and ESI Fig. S5†). This is consistent with literature data for synthetic *N*-benzoyl-l-proline methyl ester where a 4 : 1 mixture of the two rotamers was observed.^31^

### Defining key biosynthetic genes in the *scl* cluster

**Fig. 4:**
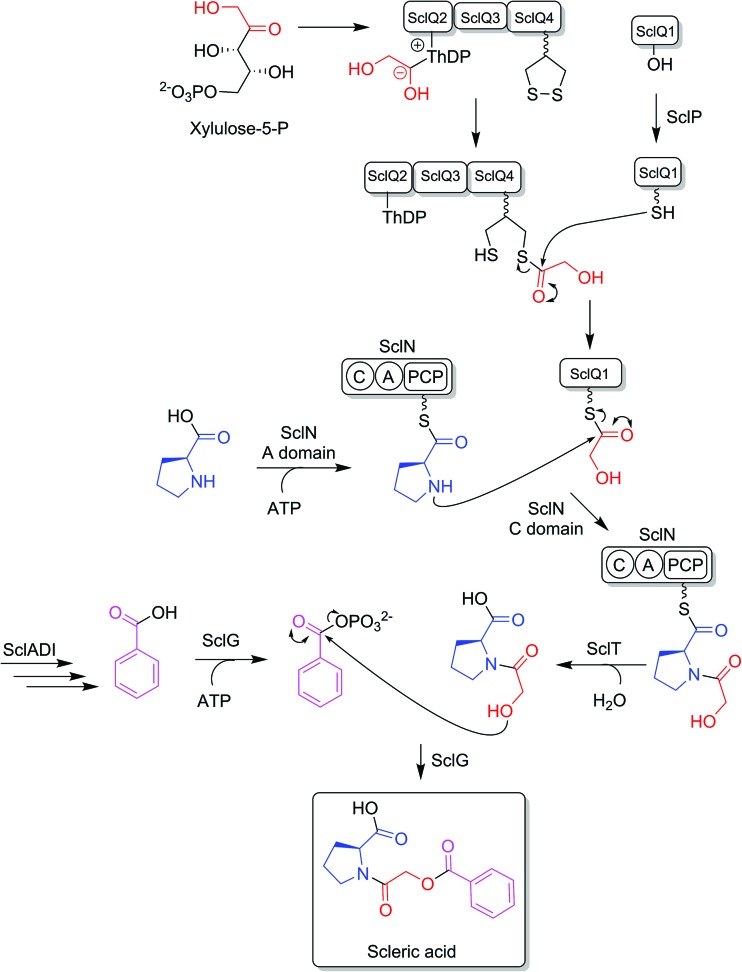
Proposed biosynthetic pathway to scleric acid.

BLASTp analyses^24^ combined with the elucidated structure for scleric acid allowed us to propose plausible functional assignments for all the biosynthetic enzymes coded in the *scl* BGC (Table 1 and Fig. 4).^32^–^34^ Three main building blocks were identified as being part of scleric acid: a glycolic acid unit (highlighted in red in Fig. 4), a benzoic acid unit (in magenta in Fig. 4) and an l-proline residue (in blue in Fig. 4). The origin of these three building blocks and the overall biosynthesis of scleric acid are discussed in detail here.

The four proteins encoded by genes *sclQ1-4* showed high homology to a set of three enzymes – QncN, QncL and QncM – from *Streptomyces melanovinaceus*. This set of enzymes has been shown to direct the biosynthesis and attachment of a C_2_-glycolicacyl unit to a non-ribosomal peptide.^34^ More specifically, SclQ2 was homologous to QncN, a thiamin diphosphate (ThDP) binding domain. SclQ3 was homologous to the first two N-terminal domains of QncL, a pyruvate dehydrogenase/transketolase pyrimidine binding domain and a transketolase C-terminal domain while SclQ4 was homologous to the last two domains of QncL, a lipoyl attachment domain and an acyltransferase catalytic domain. Lastly, SclQ1 was homologous to the acyl carrier protein (ACP) QncM. Based on the homology of SclQ1-4 with QncN, QncL and QncM, we propose that SclQ1-4 are overall responsible for converting of a ketose phosphate from the primary metabolism (such as xylulose-5-phosphate) into the activated glycolic acid unit found in scleric acid.

The three-gene cassette made of *sclA*, *sclD* and *sclI* showed high homology to genes involved in biosynthesis of a benzoic acid unit in *Streptomyces* sp. YN86.^33^ Specifically, SclA showed high similarity to the anthranilate synthase enzyme PauY18, SclD to the DAHP synthase PauY21 and SclI to the isochorismatase PauY19. Overall these three enzymes were hypothesised to be responsible for biosynthesis of the benzoyl group found in scleric acid *via* chorismate as an intermediate.

SclN was predicted to be a NRPS enzyme consisting of a single minimal elongation module: a putative, atypical condensation domain (C), an adenylation domain (A) and a peptidyl carrier protein (PCP) domain.^35^ The SclN A-domain was predicted to specifically activate l-proline, which was in accordance with the presence of a l-proline residue in scleric acid.^25^ The SclN C-domain was proposed to catalyse the amide bond formation between l-proline and glycolic acid.

Other genes putatively involved in biosynthesis and export of scleric acid and present in the *scl* cluster are: *sclT*, *sclG* and *sclE*. The thioesterase SclT is predicted to release the l-proline–oxyacetic acid intermediate from the PCP-domain of SclN. We propose that the ATP-grasp family enzyme SclG would bind and activate the benzoic acid unit produced by SclADI. That same enzyme would also promote condensation of the benzoyl unit with l-proline–oxyacetic acid, giving scleric acid. This would be exported out of the cell by the putative MFS transporter SclE.

In order to confirm the proposed involvement of the enzymes SclN, SclA and SclQ1-4 in the biosynthesis of the building blocks that make up scleric acid, we constructed gene deletion mutants in strains where the transcriptional repressor *sclM4* has also been inactivated (*S. albus*/*scl* Δ*sclM4* background). Plasmids pCm2-*sclN*, pCm2-*sclA* and pCm2-*sclQ1-4* were assembled and used to generate double mutant strains *S. albus*/*scl* Δ*sclM4* Δ*sclN*, *S. albus*/*scl* Δ*sclM4* Δ*sclA* and *S. albus*/*scl* Δ*sclM4* Δ*sclQ1-4.* Deletions were confirmed by PCR screening (ESI Fig. S3†). UHPLC-HRMS analysis revealed that production of scleric acid was abolished in *S. albus*/*scl* Δ*sclM4* Δ*sclN* and *S. albus*/*scl* Δ*sclM4* Δ*sclA* (Fig. 3a), confirming the essential role of SclN and SclA. Residual scleric acid production was detected from *S. albus*/*scl* Δ*sclM4* Δ*sclQ1-4*; this could be explained by the fact that glycolic acid is known to be produced by *Streptomyces* species in particular for the biosynthesis of *N*-glycolylmuramic acid.^36^ Addition of 5 mM glycolic acid to the culture medium of *S. albus*/*scl* Δ*sclM4* Δ*sclQ1-4* also resulted in scleric acid being produced in similar level to that observed with *S. albus*/*scl* Δ*sclM4* (ESI Fig. S16†).

The identification of key precursors in scleric acid biosynthesis was also exploited to further increase the titres of scleric acid produced by *S. albus*/*scl* Δ*sclM4*. Enriching the culture medium with 5 mM l-proline, 5 mM benzoic acid or 5 mM glycolic acid significantly increased levels of scleric acid observed upon UHPLC-HRMS analysis of the ethyl acetate extracts compared to those observed with *S. albus*/*scl* Δ*sclM4* grown on the standard supplemented minimal medium (ESI Fig. S17†). The strategy of manipulating the pathway-specific transcriptional regulatory system also makes scleric acid production not reliant on a complex culture medium. Importantly the utilisation of supplemented minimal media does significantly facilitate the isolation of the natural product of interest.

### Evidence for proposed biosynthetic intermediates

Based on the predicted function of the *scl* biosynthetic genes, we therefore proposed a putative biosynthetic route to scleric acid as shown in Fig. 4. A glycolic acid unit is believed to be produced by SclQ2-4 and loaded onto the SclQ1 ACP. SclP would convert apo-SclQ1 into holo-SclQ1 by phosphopantetheinylation. Meanwhile the adenylation domain of the NRPS SclN would activate l-proline, which could then be condensed to the glycolic acid unit through the activity of the C-domain of SclN. The l-proline–oxyacetic acid intermediate would then be released from the PCP-domain of SclN by the activity of the thioesterase SclT. The three enzymes SclADI would direct the biosynthesis of the benzoic acid moiety, which would be activated by the ATP-grasp family enzyme SclG. That same enzyme would also catalyse the condensation of the benzoyl unit with l-proline–oxyacetic acid, giving scleric acid. Lastly, scleric acid would be exported out of the cell by the putative MFS transporter SclE.

In support to this proposed pathway, we investigated by UHPLC-HRMS accumulation of the l-proline–oxyacetic acid intermediate from the ethyl acetate extracts of the scleric acid producing strain, as well as of the double mutant strains. A compound with a retention time of 3.0 minutes on C_18_ reverse phase HPLC column and an *m*/*z* value of 174.0763 [M(C_7_H_12_NO_4_) + H]^+^ (calculated *m*/*z* of 174.0761) was detected in the scleric acid producing strain *S. albus*/*scl* Δ*sclM4*, as well as in the double mutants *S. albus*/*scl* Δ*sclM4* Δ*sclQ1-4* and *S. albus*/*scl* Δ*sclM4* Δ*sclA* (ESI Fig. S18†). Consistent with the predicted function of the l-proline-activating NRPS SclN, strain *S. albus*/*scl* Δ*sclM4* Δ*sclN* did not show any accumulation of l-proline–oxyacetic acid. In order to further confirm the identity of this intermediate, which was detected from the crude extracts in amounts not sufficient for HPLC purification and subsequent NMR characterisation, a synthetic standard was prepared (see ESI Fig. S22† for a schematic representation of the synthetic route) and run alongside the crude extracts on UHPLC-HRMS. This showed the same retention time and mass spectrum as the natural product l-proline–oxyacetic acid intermediate (ESI Fig. S18†). Moreover, we grew *S. albus*/*scl* Δ*sclM4* Δ*sclN*, unable to produce scleric acid, in the presence of 5 mM l-proline–oxyacetic acid. Feeding the intermediate to the mutant strain restored production of scleric acid, as visible from UHPLC-HRMS analysis of its acidified ethyl acetate extracts (ESI Fig. S19†). This provides additional evidence that l-proline–oxyacetic acid is a true intermediate in scleric acid biosynthesis. It also suggests that the l-proline–oxyacetic acid is released from the C-domain of SclN prior to SclG catalysing its condensation with the benzoyl group, in accordance with the order of reactions proposed in Fig. 4.

### Biological activity of scleric acid

The antimicrobial activity of scleric acid was assessed. Inhibitory activity against representatives of the ESKAPE panel of pathogens: *Enterococcus faecium*, *Staphylococcus aureus*, *Klebsiella pneumoniae*, *Acinetobacter baumannii*, *Pseudomonas aeruginosa*, and *Enterobacter cloacae* was first screened but all strains appeared to be resistant to scleric acid, giving no observable MIC (ESI Table S6†).

Scleric acid was then tested for a broader range of pharmaceutically relevant bioactivities through the Eli Lilly Open Innovation Drug Discovery (OIDD) Program. In a single point (20 μM) primary assay, scleric acid showed moderate antibacterial activity against *Mycobacterium tuberculosis* (H37Rv), exhibiting a 32% inhibition on the growth of this strain.

Scleric acid showed inhibitory activity on the cancer-associated metabolic enzyme nicotinamide *N*-methyltransferase (NNMT), the overexpression of which is known to contribute to tumorigenesis.^37^ NNMT catalyses the transfer of a methyl group from *S*-adenosyl-l-methionine (SAM) to nicotinamide, generating *S*-adenosyl-l-homocysteine (SAH) and 1-methylnicotinamide (MNAN).^37^ Scleric acid showed, on a concentration response curve assay, IC_50_ of 178.0 μM (NNMT MNAN) and 186.6 μM (NNMT SAH) (ESI Fig. S4†).

## Conclusions

Specialised metabolites from actinomycete bacteria are one of the most valuable sources of novel antibiotics, as well as of other useful bioactive compounds employed in various fields, from human medicine to crop protection. High-throughput sequencing of bacterial genomes/metagenomes has become quick and inexpensive, and is unearthing a myriad of putative gene clusters that are awaiting to be characterised and exploited. In this study, we have demonstrated that a cryptic gene clusters from a genetically intractable actinomycete could be successfully exploited for characterising the biosynthetic pathway it encodes as well as the resulting natural product. In principle, the same approach could be used with metagenomic DNA. Our strategy first relied on the bioinformatics identification and selection of cryptic gene clusters containing a characterised regulatory system (Fig. 1). A DNA fragment containing the entire gene cluster was then captured *via* TAR cloning and introduced in the genome of a validated heterologous host. Expression of the biosynthetic genes was then triggered through CRISPR/Cas9-mediated editing of the key *mmyR*-like transcriptional repressor. Analytical chemistry procedures were then undertaken to identify, isolate and characterise the new natural product, overproduced in the heterologous host. Finally, subsequent rounds of CRISPR/Cas9-mediated gene deletions targeted putative biosynthetic genes and afforded mutant strains. In turn, a biosynthetic route to the novel natural product could be proposed. This approach was validated by the discovery of (2-(benzoyloxy)acetyl)-l-proline, named scleric acid, from the genome of the soil-dwelling filamentous bacterium *Streptomyces sclerotialus* NRRL ISP-5269. Based on the predicted function of the Scl enzymes and the detection of a key intermediate from culture extracts of selected strains a plausible biosynthetic route to scleric acid was proposed (Fig. 4). In addition to the novel biochemistry this biosynthetic pathway offers, scleric acid has been shown to exhibit moderate antibacterial activity against *M. tuberculosis*, as well inhibition on the cancer-associated enzyme nicotinamide *N*-methyltransferase (NNMT). We are currently investigating scleric acid bioactivity further through the Eli Lilly Open Innovation Drug Discovery (OIDD) Program.

The widespread presence of orthologues of the methylenomycin regulatory genes among actinomycete genomes (ESI Table S4†) revealed that the approach described herein might be very promising for the discovery and characterisation of novel natural products, and therefore, of novel biocatalysts. Comparative genomics analyses also indicated that there is no apparent correlation between the presence of the regulatory cassette we targeted and the type of natural products that they regulate production of – both in relation to biosynthesis and bioactivity – methylenomycin,^9^ gaburedins^12^ and scleric acid being examples of natural products characterised so far.

In conclusion, beyond the discovery of this specialised metabolite, we strongly believe that targeting conserved pathway-specific regulatory elements, as opposed to mining BGCs encoding defined enzymatic machineries (*i.e.* PKS, NRPS), will lead to the identification and characterisation of microbial natural products assembled by truly novel types of biocatalysts.

## Materials and methods

### Bioinformatics analysis

The whole genome sequence of *Streptomyces sclerotialus* NRRL ISP-5269 was downloaded from the Genomes Online Database of the JGI Portal (U.S. Department of Energy, ; https://gold.jgi.doe.gov/, GOLD Project ID Gp0187859). Contig43 of the genome contained the *scl* gene cluster object of this study. A combination of AntiSMASH^23^ and manual BLASTp^24^ analyses allowed to define the putative borders of the *scl* cluster and predict the function of the encoded enzymes. The website for PKS/NRPS analysis from the University of Maryland was used for domain prediction of SclN.^25^ SerialCloner 2.6.1 (SerialBasics) was used for DNA sequence analysis and plasmid design. Multigene BLAST^21^ was used to search for gene clusters that included homologues of the methylenomycin regulatory genes from *S. coelicolor* A3(2) and allowed to pinpoint the *scl* BGC from *S. sclerotialus* NRRL ISP-5269. ClusterTools was used to search orthologues of the proteins involved in regulation of methylenomycin production in other actinomycetes (ESI Table S4†), as well as orthologues of the *scl* cluster proteins in other actinomycetes (ESI Table S5†).^22^

### Reagents

All chemicals were purchased from Sigma-Aldrich, unless otherwise stated. Phusion DNA polymerase, as well as all restriction endonucleases, T4 DNA ligase, shrimp alkaline phosphatase (rSAP) and Gibson Assembly cloning kit, were purchased from New England Biolabs. Zymolyase 20T was purchased from MP Biomedicals, 5-fluoroorotic acid (5-FOA) was purchased from Thermo Fisher Scientific. Primers for PCR amplification were purchased from Sigma-Aldrich (see ESI Table S3† for a list of oligonucleotides used in this study).

### Culturing and engineering of microorganisms

*Streptomyces sclerotialus* NRRL ISP-5269 was obtained from JCM (Japan Collection of Microorganisms, culture collection number 4828^T^) (see ESI Table S1† for a list of strains used in this study). *Streptomyces albus* J1074 and *Streptomyces coelicolor* M1152 were used for heterologous expression. All *Streptomyces* strains were grown on soya flour mannitol (SFM) agar medium (20 g L^–1^ soya flour, 20 g L^–1^ mannitol, 20 g L^–1^ agar), with appropriate antibiotic selection upon insertion of plasmid DNA (50 μg mL^–1^ apramycin when transformed with pCRISPomyces-2-derived plasmids; 25 μg mL^–1^ kanamycin when transformed with pCAP03-derived plasmids; 25 μg mL^–1^ nalidixic acid on the first round of subculture after intergeneric conjugation). *E. coli* ET12567 and ET12567/pUB307 were used for the purpose of intergeneric tri-parental conjugation. One Shot TOP10 chemically competent *E. coli* cells (Thermo Fisher Scientific) were used for cloning and storage of plasmid DNA. All *E. coli* strains were grown on lysogeny broth (LB) medium (10 g L^–1^ tryptone, 5 g L^–1^ yeast extract, 10 g L^–1^ NaCl) or LB agar medium (same as LB medium, with 15 g L^–1^ agar), with appropriate antibiotic selection (50 μg mL^–1^ apramycin when transformed with pCRISPomyces2-derived plasmids, 25 μg mL^–1^ chloramphenicol to maintain the *dam* mutation in *E. coli* ET12567, 25 μg mL^–1^ kanamycin either to maintain helper plasmid pUB307 or after insertion of pCAP03-derived plasmids).

*S. cerevisiae* VL6-48N was used for TAR cloning and grown on yeast extract peptone (YPD) broth (5 g L^–1^ yeast extract, 10 g L^–1^ peptone, 2% w/v glucose) or YPD agar (same as YPD, with 15 g L^–1^ agar). Purification of genomic DNA from *S. sclerotialus* was performed from a 100 mL liquid culture by phenol-chloroform extraction.^13^ The *scl* gene cluster was captured using TAR cloning.^26^ Assembly of plasmid pCAP03-*scl* was performed following the procedure described by Moore and colleagues; pCAP03 was a gift from Bradley Moore (Addgene plasmid # 69862) (see ESI Table S2† for a list of plasmids used in this study).^27^ For this purpose String DNA fragments (Thermo Fisher Scientific) were ordered to include 60-bp hooks homologous to either side of the *scl* cluster (ESI Table S2†) and introduced into pCAP03 *via* Gibson Assembly (New England Biolabs). The identity of the captured cluster was confirmed by PCR amplification and restriction digestion (ESI Fig. S1†). Insertion of the *scl* gene cluster in the genome of the heterologous hosts *S. albus* and *S. coelicolor* was accomplished *via* intergeneric tri-parental conjugation following the protocol described by Moore and colleagues.^27^ CRISPR/Cas9-based engineering of *S. albus* strains was performed using plasmids pCm2-*sclM4*, pCm2-*sclN*, pCm2-*sclA* and pCm2-*sclQ1-4*. Golden Gate Assembly was first performed to insert the specific sgRNAs into the backbone pCm2 plasmid, then Gibson Assembly was used to include 800-bp homologous recombination arms, all following the procedure described by Zhao and colleagues; pCRISPomyces-2 was a gift from Huimin Zhao (Addgene plasmid # 61737).^15^ Clearance of temperature sensitive plasmids based on pCm2 was achieved by culturing the mutant strains on SFM agar medium non-selectively at 39 °C.

### Identification, isolation and structure elucidation of scleric acid

*S. albus* strains were cultured for 5 days at 30 °C on supplemented solid minimal (SM) medium (2 g L^–1^ casaminoacids, 8.68 g L^–1^ TES buffer, 15 g L^–1^ agar; after autoclaving, and just before use, 10 mL of 50 mM NaH_2_PO_4_ + K_2_HPO_4_, 5 mL of 1 M MgSO_4_, 18 mL of 50% w/v glucose and 1 mL of trace element solution [0.1 g L^–1^ each of ZnSO_4_·7H_2_O, FeSO_4_·7H_2_O, MnCl_2_·4H_2_O, CaCl_2_·6H_2_O and NaCl] were added) as described by Hopwood and colleagues.^13^ Ethyl acetate was added in equal volume to the volume of SM medium used, and acidified to pH 3 by the addition of 37% HCl. The ethyl acetate layer was removed and evaporated under reduced pressure. The remaining residue was dissolved in 500 μL of 50 : 50 (v/v) HPLC grade methanol/water for UHPLC-HRMS analysis. For purification of scleric acid, 2 L of SM culture medium was used for organic extractions, and the remaining residue after evaporation of ethyl acetate was dissolved in 10 mL 50 : 50 HPLC grade methanol/water for silica column pre-purification.

UHPLC-HRMS analyses were carried out with 20 μL of prepared extracts injected through a reverse phase column (Zorbax Eclipse Plus C18, size 2.1 × 100 mm, particle size 1.8 μm) connected to a Dionex 3000RS UHPLC coupled to Bruker Ultra High Resolution (UHR) Q-TOF MS MaXis II mass spectrometer with an electrospray source. Sodium formate (10 mM) was used for internal calibration and a *m*/*z* scan range of 50–1500 was used with a gradient elution from 95 : 5 solvent A/solvent B to 0 : 100 solvent A/solvent B over 10 minutes. Solvents A and B were water (0.1% HCOOH) and acetonitrile (0.1% HCOOH), respectively.

Pre-purification of crude extract containing scleric acid was performed using flash chromatography. A column was loaded with C18-reversed phase silica gel, preconditioned with one volume of methanol, activated with one volume of solvent B (0.045% v/v trifluoroacetic acid in acetonitrile), and equilibrated with two volumes of solvent A (0.045% v/v trifluoroacetic acid in water). Crude extract was loaded onto the column. Compounds were then eluted with five different consecutive solvent systems: two volumes of 20 : 80 solvent B/solvent A, two volumes of 40 : 60 solvent B/solvent A, two volumes of 50 : 50 solvent B/solvent A, two volumes of 60 : 40 solvent B/solvent A and two volumes of 80 : 20 solvent B/solvent A. Fractions were collected throughout the elution steps, evaporated under reduced pressure and dissolved in 500 μL of 50 : 50 (v/v) HPLC grade methanol/water for UHPLC-HRMS analysis. Fractions containing scleric acid were combined and used for HPLC purification.

Reverse-phase HPLC was performed using a Zorbax XBD-C18 column (212 × 150 mm, particle size 5 μm) connected to an Agilent 1200 HPLC equipped with a binary pump and DAD detector. Solvent A: 0.1% TFA water, solvent B: 0.1% TFA in acetonitrile, 5% B to 95% B in 45 min. Retention time compound 1: 29.7 min, retention time compound 2 (scleric acid): 34.4 min. Gradient elution was used (solvent A: water with 0.1% HCOOH, solvent B: methanol) with a flow rate of 10 mL min^–1^. Fractions were collected by time or absorbance at 210 nm using an automated fraction collector. The fractions collected containing scleric acid were pooled, methanol removed under reduce pressure and scleric acid was re-extracted from the remaining water (2 × 50 mL ethyl acetate). The ethyl acetate was removed under reduced pressure and the sample re-dissolved in deuterated methanol for NMR analysis.

### MIC testing

The susceptibility of bacterial strains was investigated in collaboration with the Warwick Antimicrobial Screening Facility in a 96-well plate experiment, according to the Clinical & Laboratory Standards Institute (CLSI) guidelines (M7-A9 2012). Scleric acid was diluted to a concentration of 7.5 mg mL^–1^ in 25% DMSO in distilled water. To further prevent toxicity effects from DMSO in MIC testing, 27 μL of the natural product stock was combined with 173 μL of cation adjusted Muller-Hinton broth to a final concentration of 1024 μg mL^–1^ of compound in 200 μL with 3% DMSO. This was then further doubling diluted throughout the MIC. Meropenem and cefoxitin were used as positive controls during MIC testing.

## Conflicts of interest

There are no conflicts to declare.

## Supplementary Material

Supplementary information
