# Supplementary material for: Triggering the expression of a silent gene cluster from genetically intractable bacteria results in scleric acid discovery
Source: Chem Sci. 2018 Oct 19;10(2):453–63. doi: 10.1039/c8sc03814g (PMC6335953; doi:10.1039/c8sc03814g)
Supplement: Supplementary file 1 [file SC-010-C8SC03814G-s001.pdf]

Electronic Supplementary Material (ESI) for Chemical Science.
This journal is © The Royal Society of Chemistry 2018

ELECTRONIC SUPPLEMENTARY INFORMATION

Triggering the expression of a silent gene cluster from genetically intractable bacteria results in scleric acid discovery

Fabrizio Alberti,*[a,b] Daniel J. Leng,[b] Ina Wilkening,[b] Lijiang Song,[b] Manuela Tosin[b] and Christophe Corre*[a,b]

[a] *Warwick Integrative Synthetic Biology Centre and School of Life Sciences, University of Warwick, Coventry, CV4 7AL, UK.*

[b] *Department of Chemistry, University of Warwick, Coventry, CV4 7AL, UK.*

* Corresponding authors: F.Alberti@warwick.ac.uk, C.Corre@warwick.ac.uk

Table of Contents

1. Biology supplementary methods and results 3
Table S1. List of strains used in this study. 3
Table S2. List of plasmids used in this study. 3
Table S3. List of oligonucleotides used in this study. 4
Table S4. Orthologous genes/proteins to those found in the methylenomycin regulatory system from *S. coelicolor* A3(2) identified using clusterTools.[8] 5
Table S5. Summary of the number of biosynthetic gene cassettes orthologous to those found in the *scl* cluster, identified using clusterTools.[8] 6
Figure S1. Confirmed identity of the captured *scl* cluster. 6
Figure S2. CRISPR/Cas9-guided deletion generated on *sclM4*. 7
Figure S3. CRISPR-Cas9-guided deletions generated on key putative biosynthetic genes of the gene cluster. 7
Figure S4. Determination of IC_{50} for scleric acid against Nicotinamide *N*-methyltransferase (NNMT). 8
Table S6. MIC values determined for scleric acid against ESKAPE pathogenic bacterial strains. 8
2. Chemistry supplementary results 9
2.1. Characterisation of scleric acid and *L*-proline-oxyacetic acid intermediate 9
Figure S5. NMR assignment (700 MHz, CD_3OD) of scleric acid (m/z 278.1020, $C_{14}H_{15}NO_5$). 9
Figure S6. ^{1}H-NMR spectrum (700 MHz, CD_3OD) of scleric acid isolated from *S. albus/scl* Δ*sclM4* (top panel) and synthetic scleric acid (lower panel). 10
Figure S7. COSY spectrum (700 MHz, CD_3OD) of scleric acid. 10
Figure S8. HSQC spectrum (700 MHz, CD_3OD) of scleric acid. 11
Figure S9. HMBC spectrum (700 MHz, CD_3OD) of scleric acid. 11
Figure S10. LC-MS analyses of proline residues derivatised with Marfey's reagent for absolute stereochemistry determination.[9] 12
Figure S11. ^{1}H-NMR spectrum (500 MHz, CD_3OD) of the synthetic scleric acid analogue 2-((benzoyl-*L*-prolyl)oxy)acetic acid. 13
Figure S12. COSY spectrum (500 MHz, CD_3OD) of the synthetic scleric acid analogue 2-((benzoyl-*L*-prolyl)oxy)acetic acid. 13
Figure S13. HSQC spectrum (500 MHz, CD_3OD) of the synthetic scleric acid analogue 14
Figure S14. HMBC spectrum (500 MHz, CD_3OD) of the synthetic scleric acid analogue 2-((benzoyl-*L*-prolyl)oxy)acetic acid. 14
Figure S15. ^{13}C spectrum (125 MHz, CD_3OD) of the synthetic scleric acid analogue 2-((benzoyl-*L*-prolyl)oxy)acetic acid. 15

Figure S16. Restored production of scleric acid in *S. albus/scl* Δ*sclM4* Δ*Q1-4* strain upon feeding with glycolic acid....15
Figure S17. Scleric acid increment in precursor enriched medium detected with UHPLC-HRMS..16
Figure S18. Detection of the *L*-proline-oxyacetic acid intermediate with UHPLC-HRMS....16
Figure S19. Detection of scleric acid by UHPLC-HRMS in *S. albus/scl* Δ*sclM4* Δ*sclN* fed with *L*-proline-oxyacetic acid....17
2.2. Synthetic Chemistry 17
2.2.1.Synthesis of scleric acid....17
Figure S20. Schematic representation of synthetic route to scleric acid. 17
2.2.2.Synthesis of the scleric acid structural isomer....20
Figure S21. Schematic representation of synthetic route to the scleric acid structural isomer 2-((benzoyl-prolyl)oxy)acetic acid. 20
2.2.3.Synthesis of the *L*-proline-oxyacetic acid intermediate....21
Figure S22. Schematic representation of synthetic route to *L*-proline-oxyacetic acid. 21
References....23

1. Biology supplementary methods and results

Table S1. List of strains used in this study.

Name	Description	Reference
***Streptomyces sclerotialus* NRRL ISP-5269**	Strain from which the *scl* cluster was cloned	1
***Streptomyces albus* J1074**	Used as heterologous host	2
***Streptomyces coelicolor* M1152**	Used as heterologous host	3
Streptomyces albus/scl	Strain engineered to contain the *scl* cluster	This study
Streptomyces coelicolor* M1152*/scl	Strain engineered to contain the *scl* cluster	This study
***Streptomyces albus*/pCAP03**	Control strain engineered to contain the empty plasmid pCAP03	This study
***Streptomyces coelicolor* M1152/pCAP03**	Control strain engineered to contain the empty plasmid pCAP03	This study
***Escherichia coli* ET12567**	Methylation deficient strain used for intergeneric conjugation	4
***Escherichia coli* ET12567/pUB307**	Strain with self-transmissible plasmid that mobilises other plasmids in trans for DNA transfer into *Streptomyces* hosts	5
Streptomyces albus/scl* Δ*sclM4	Strain with inactivated transcriptional repressor, producer of scleric acid	This study
Streptomyces albus/scl* Δ*sclM4* Δ*sclN	Strain with inactivated transcriptional repressor and NRPS	This study
Streptomyces albus/scl* Δ*sclM4* Δ*sclA	Strain with inactivated transcriptional repressor and Anthranilate synthase	This study
Streptomyces albus/scl* Δ*sclM4* Δ*sclQ1-4	Strain with inactivated transcriptional repressor and glycolic acid biosynthesis genes	This study
Staphylococcus aureus	Members of the ESKAPE pathogenic panel used for antimicrobial activity assay	ATCC BAA-1717
Enterobacter cloacae		NCTC 13405
Acinetobacter baumanii		ATCC 19606
Pseudomonas aeruginosa		ATCC 27853
Enterococcus faecium		ATCC 12202
Klebsiella pneumoniae		ATCC 700603
***Escherichia coli* TOP10**	Host strain used for cloning	Invitrogen
S. cerevisiae VL6-48N	Used for *in vivo* capturing of the *scl* cluster through homologous recombination	ATCC MYA-3666

Table S2. List of plasmids used in this study.

**Name**	**Use**	**Selectable marker**	**Reference**
pUB307	Self-transmissible plasmid that mobilises other plasmids in trans for DNA transfer into *Streptomyces* hosts	Kan^R	5
pCAP03	Capture of gene clusters upon insertion of hooks for homologous recombination	Kan^R	6
pCRISPomyces2	Backbone plasmid for CRISPR-Cas9-mediated genome editing of actinomycetes upon insertion of synthetic guide RNAs (sgRNAs) and homologous recombination arms (HR arms)	$Apra^R$	7
pCAP03-scl[a]	Capture of the *scl* gene cluster from *S. sclerotialus*	Kan^R	This study
pCm2-sclM4[b]	Deletion of *sclM4*	$Apra^R$	This study
pCm2-sclN[c]	Deletion of *sclN*	$Apra^R$	This study
pCm2-sclA[d]	Deletion of *sclA*	$Apra^R$	This study
pCm2-sclQ1-4[e]	Deletion of *sclQ1-4*	$Apra^R$	This study

[a]Left hook for homologous recombination:
AAGATGCCGAGCTGGCTGCGGGATTCGCGCACCGCGATGAAGGAGACCGGCAAGGCCCTG
Right hook for homologous recombination:
CGTCGCGATCATGGTGGGCAACACCGAGTGGCCCAAGTGGGAGAAGGTCATGGCGGCCGA
[b]sgRNA: GGTGCTGGCGAACCCGAGGG
[c]sgRNA1: TCCGGATGGTGCGAGCGCAG, sgRNA2: CCGAGACGGTCGCGGCGGGA
[d]sgRNA1: CCTCGACCGACCAGTGCTCC, sgRNA2: CCGTGATGACCGCTTCATCG
[e]sgRNA1: ACGCCGCTCGGTACCGTCGC, sgRNA2: GGGCATCACGCACGGGTCCC

Table S3. List of oligonucleotides used in this study.

Name	Sequence	Use
pCm2-sclM4_protosp FF	acgcGGTGCTGGCGAACCCGAGGG	Insertion of protospacer in pCm2-sclM4
pCm2- sclM4_protosp RR	aaacCCCTCGGGTTCGCCAGCACC	
pCm2-sclM4 LA FF	tgccgccgggcgtttttatGCCGCTCTCGAAGTCGAGGACGGCG	Amplification of homologous recombination left arm for pCm2-sclM4
pCm2-sclM4 LA RR	CTGCCGCGGAGTTGCACCAATCTCCAGGTGGTGGCG	
pCm2-sclM4 RA FF	CACCACCTGGAGATTGGTGCAACTCCGCGGCAGCCGC GTCCAAG	Amplification of homologous recombination right arm for pCm2-sclM4
pCm2-sclM4 RA RR	ctttttacggttcctggcctCCGCAGGCCGGAGTTGCGCCAGGTG	
Sequencing sclM4 HR FF	CGGGCATCTGATCGCCCTGGTG	PCR amplification of *sclM4* to assess deletion from pCm2-sclM4
Sequencing sclM4 HR RR	TCCGGTGGCAAGTACCTCCAGC	
pCm2-sclN LA FF	tcggttgccgccgggcgtttttatCGTGCCACGCACGGACGT	Amplification of homologous recombination left arm for pCm2-sclN
pCm2-sclN LA RR	GGATGTGGTGGTGCTCATCCGGAGCGCAGGCTCGTG	
pCm2-sclN RA FF	CACGAGCCTGCGCTCCGGATGAGCACCACCACATCC	Amplification of homologous recombination right arm for pCm2-sclN
pCm2-sclN RA RR	gcggcctttttacggttcctggcctCTGGTCGGTCGAGGACCC	
Sequencing sclN HR FF	GTTCATGCGGACTGGATAC	Amplification of *sclN* to assess deletion from pCm2-sclN
Sequencing sclN HR RR	GGTGGATCAGGGCGAAAG	
pCm2-sclA LA FF	tcggttgccgccgggcgtttttatGGTCGCGGCGGGACGGGA	Amplification of homologous recombination left arm for pCm2-sclA
pCm2-sclA LA RR	CATGGCCGTATTGATCACGGAGCCCGTCTGTGTGTG	
pCm2-sclA RA FF	CACACACAGACGGGCTCCGTGATCAATACGGCCATG	Amplification of homologous recombination right arm for pCm2-sclA
pCm2-sclA RA RR	gcggcctttttacggttcctggcctGCGAGCAGCTCGTCGCGT	
Sequencing sclA HR FF	ACCGGTCCTCGACTTCTTC	Amplification of *sclA* to assess deletion from pCm2-sclA
Sequencing sclA HR RR	GTTGGGGTCGAGGCGTTC	
pCm2-sclQ1-4 LA FF	tcggttgccgccgggcgtttttatCGTACGGTCCACCCCGCC	Amplification of homologous recombination left arm for pCm2-sclQ1-4
pCm2-sclQ1-4 LA RR	CTCTGGAGTGTCTGACGCGGCCGGTGCTCCGCCGTG	
pCm2-sclQ1-4 RA FF	CACGGCGGAGCACCGGCCGCGTCAGACACTCCAGAG	Amplification of homologous recombination right arm for pCm2-sclQ1-4
pCm2-sclQ1-4 RA RR	gcggcctttttacggttcctggcctACGGGCCGGTGGCGCACT	
Sequencing sclQ1-4 HR FF	TTCCAGGAGGTCACCGAC	Amplification of *sclQ1-4* to assess deletion from pCm2-sclQ1-4
Sequencing sclQ1-4 HR RR	GTCGACATCAGTTGGGACG	
Screening sclM4 FF	TTGGTACACACTGTCGCTGTCAC	Amplification of *sclM4* to assess capturing of *scl* cluster
Screening sclM4 RR	TAAGGAACCACGGATATGGTCAAAC	

Table S4. Orthologous genes/proteins to those found in the methylenomycin regulatory system from *S. coelicolor* A3(2) identified using clusterTools.[8]

#	Bacterial strain	Accession ID	Start	End	Size (nt)	BLAST Similarity Score	MmfR	MmyR	MmfL	MmfP	MmfH
1	*Streptomyces* sp. S10(2016)	NZ_CP015098.1	4782325	4786837	4513	2.0723	WP_062928109.1; WP_062928113.1	WP_062928113.1; WP_062928109.1	WP_062928110.1	WP_062928112.1	WP_062928111.1
2	*Streptacidiphilus melanogenes* strain NBRC 103184	NZ_BBPP01000016.1	12874	17454	4581	1.9478	WP_052434417.1; WP_052434418.1	WP_052434418.1; WP_052434417.1	WP_042383066.1	WP_042383065.1	WP_042383067.1
3	*Streptomyces roseoverticillatus* strain NRRL B-3500 contig22.1	NZ_JOFL01000022.1	45931	50919	4989	1.9367	WP_030368767.1; WP_052393004.1	WP_052393004.1; WP_030368767.1	WP_030368766.1	WP_052393003.1	WP_052393018.1
4	*Streptomyces kanamyceticus* strain NRRL B-2535 B-2535_contig_135	NZ_LIQU01000135.1	29180	42345	13166	1.881	WP_055547507.1; WP_055547490.1	WP_055547490.1; WP_055547507.1	WP_055547484.1	WP_063806085.1; WP_055547466.1	WP_055547486.1
5	*Kitasatospora mediocidica* KCTC 9733 BS80DRAFT_unitig_3_quiver.1_C	NZ_JQLN01000001.1	344626	354245	9620	1.7353	WP_035791765.1; WP_051965634.1	WP_051965634.1; WP_035791765.1	WP_051965635.1	WP_035791755.1	WP_063771887.1
6	*Streptomyces griseoplanus* strain NRRL B-3064 B3064_contig_350	NZ_LIQR01000350.1	1458	5768	4311	1.7252	WP_055589482.1; WP_055589478.1	WP_055589478.1; WP_055589482.1	WP_055589481.1	WP_063796061.1	WP_055589480.1
7	*Streptomyces pluripotens* strain MUSC 135	NZ_JTDH01000125.1	97777	102368	4592	1.6047	WP_039654680.1; WP_039654807.1	WP_039654807.1; WP_039654680.1	WP_063837895.1	WP_043433946.1	WP_039654677.1
8	*Streptomyces roseochromogenus* subsp. oscitans DS 12.976 chromosome	NZ_CM002285.1	5661730	5668353	6624	1.5553	WP_051430295.1; WP_023549830.1	WP_023549830.1; WP_051430295.1	WP_023549823.1	WP_031225671.1	M878_RS74185
9	*Streptacidiphilus rugosus* AM-16 BS83DRAFT_scf7180000000012_quiver.4_C	NZ_JQMJ01000004.1	70841	117141	46301	1.5077	WP_063774329.1; WP_051945291.1; WP_051942861.1	WP_051942861.1; WP_063774329.1; WP_051945291.1	WP_051942867.1; WP_037603352.1	WP_037603372.1	WP_051942859.1; WP_051942858.1
10	*Streptomyces venezuelae* ATCC 10712 complete genome	NC_018750.1	4526411	4547575	21165	1.4938	WP_015035379.1; WP_015035398.1; WP_051025910.1	WP_051025910.1; WP_015035379.1	WP_051025909.1	WP_015035382.1	WP_015035385.1
11	*Streptomyces vietnamensis* strain GIM4.0001	NZ_CP010407.1	4824251	4850159	25909	1.4671	WP_041130598.1; WP_052499245.1	WP_052499240.1; WP_041130598.1	WP_052499239.1	WP_041130599.1	WP_052499241.1
12	*Streptacidiphilus albus* JL83 BS75DRAFT_unitig_0_quiver.1_C	NZ_JQML01000001.1	5289664	5324448	34785	1.377	WP_034089620.1; WP_052069629.1; WP_034089636.1	WP_052069629.1; WP_034089620.1	WP_052069631.1	WP_052069630.1	WP_042437474.1
13	*Kitasatospora cheerisanensis* KCTC 2395 scaffold00001	NZ_KK853997.1	1382622	1389383	6762	1.2906	WP_035870914.1; WP_051652813.1	WP_051652813.1; WP_035870914.1	WP_051652810.1	WP_051652809.1	KCH_RS38560
14	*Streptomyces avermitilis* MA-4680 = NBRC 14893 DNA	NC_003155.5	2764349	2768853	4505	1.2867	WP_010983710.1; WP_010983708.1	WP_010983708.1	WP_010983709.1	WP_010983706.1	WP_010983707.1

Table S5. Summary of the number of biosynthetic gene cassettes orthologous to those found in the *scl* cluster, identified using clusterTools.[8]

Proteins query	Number of orthologues found
SclQ1-4	8
SclN and SclT	19
SclN, SclT and SclQ1	0
SclA, SclI and SclD	46
SclG and SclA	1
SclG, SclI	0
SclG, SclD	0

a **b** **c**

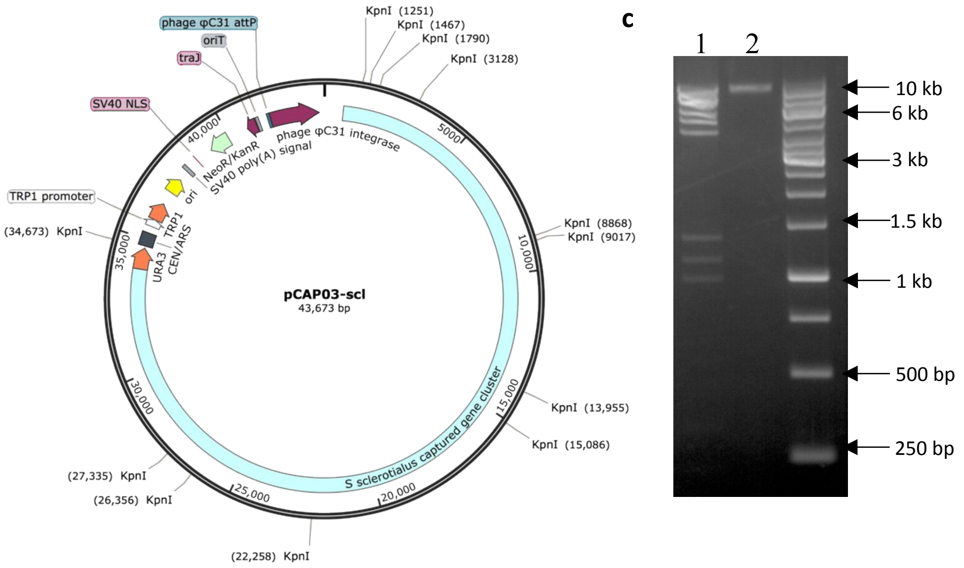

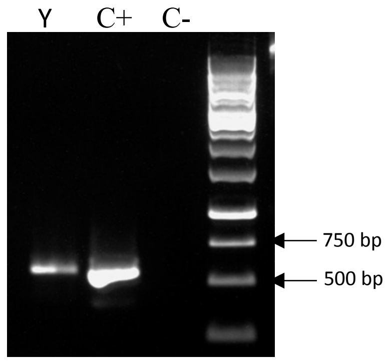
Y
C+
C-
750 bp
500 bp

Figure S1. Confirmed identity of the captured *scl* cluster.
(**a**) PCR amplification of the *sclM4* gene from yeast colony after TAR cloning (Y), genomic DNA of *S. sclerotialus* (C+) and negative control (C-). Expected amplicon size: 549 bp. (**b**) Plasmid map of pCAP03-scl. (**c**) *Kpn*I Restriction digestion of plasmids purified from *E. coli*: pCAP03-scl (1) and pCAP03 (2). Expected restriction fragments for pCAP03-scl: 10,251 bp; 7,338 bp; 7,172 bp; 5,740 bp; 4,938 bp; 4,098 bp; 1,338 bp; 1,131 bp; 979 bp, 323 bp; 216 bp; 149 bp. Expected restriction fragment for pCAP03: 11,187 bp.

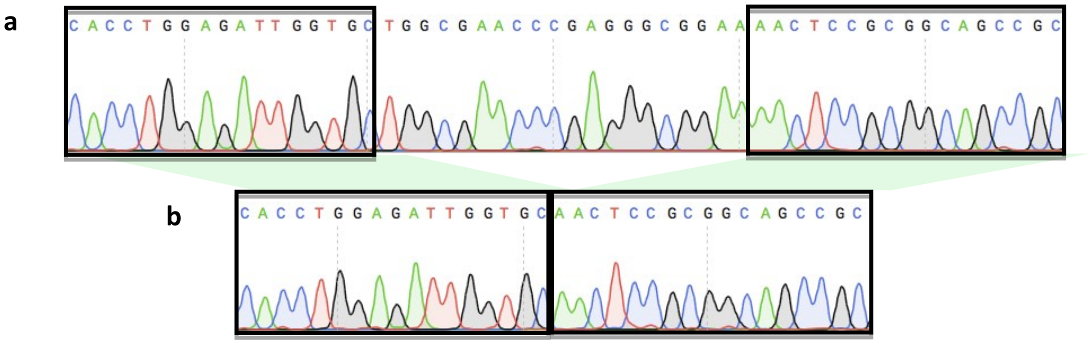

Figure S2. CRISPR/Cas9-guided deletion generated on *sclM4*.
Alignment of sequencing chromatograms showing the 20-bp short deletion at the 5' of the *sclM4* gene: (**a**) native sequence in *S. albus/scl*, (**b**) mutated sequence after 20-bp deletion with CRISPR/Cas9 in *S. albus/scl* Δ*sclM4*.

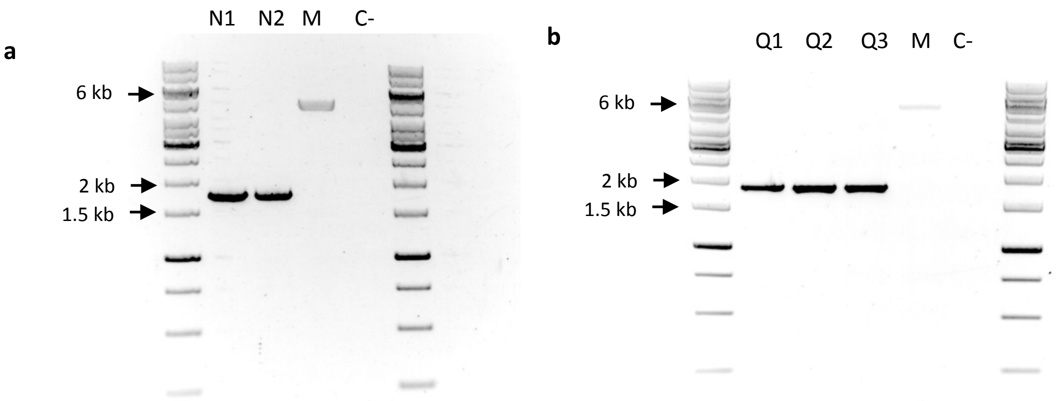

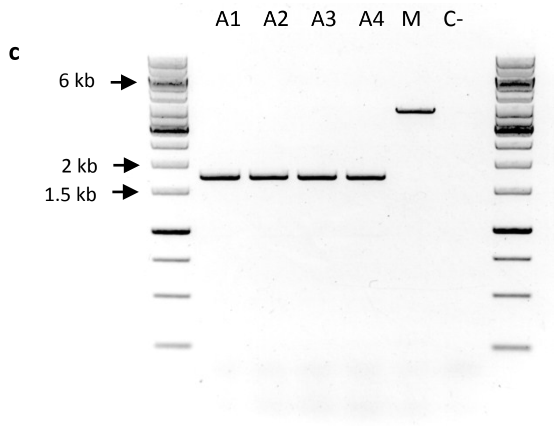

Figure S3. CRISPR-Cas9-guided deletions generated on key putative biosynthetic genes of the gene cluster.
(**a**) Deletion of *sclN* gene; expected amplicon from *S. albus*/*scl* Δ*M4* (M): 4,995 bp; expected amplicon from *S. albus*/*scl* Δ*sclM4* Δ*sclN* (N1, N2): 1,747bp. (**b**) Deletion of *sclQ1-4* operon; expected amplicon from *S. albus*/*scl* Δ*sclM4* (M): 5,722 bp; expected amplicon from *S. albus*/*scl* Δ*M4* Δ*sclQ1-4* (Q1, Q2, Q3): 1,699 bp. (**c**) Deletion of *sclA* gene; expected amplicon from *S. albus*/*scl* Δ*sclM4* (M): 4,643 bp; expected amplicon from *S. albus*/*scl* Δ*sclM4* Δ*sclA* (A1, A2, A3): 1,676 bp.

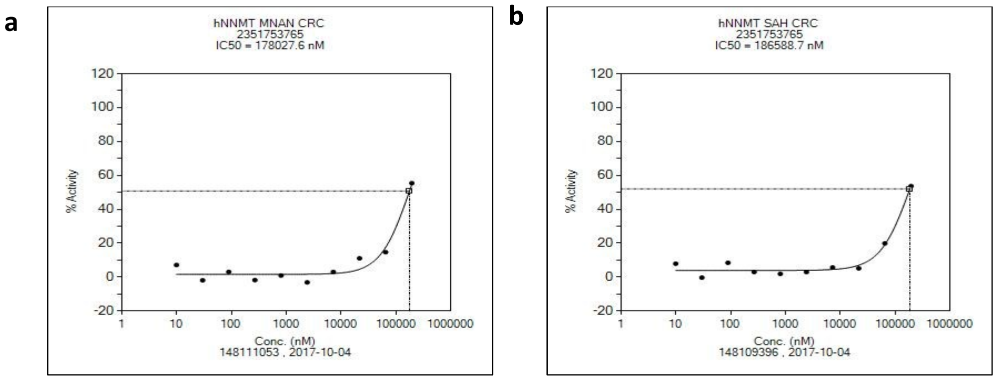

Figure S4. Determination of IC_{50} for scleric acid against Nicotinamide *N*-methyltransferase (NNMT). (**a**) *S*-adenosyl-*L*-homocysteine (SAH) concentration response curve assay; (**b**) 1-methylnicotinamide (MNAN) concentration response curve assay.

Table S6. MIC values determined for scleric acid against ESKAPE pathogenic bacterial strains.

Bacterial strain	MIC (µg/ml)
***Staphylococcus aureus* USA300**	>1024
***Enterobacter cloacae* NCTC 13405**	>1024
***Acinetobacter baumanii* ATCC 19606**	>1024
***Pseudomonas aeruginosa* ATCC 27853**	>1024
***Enterococcus faecium* ATCC 12202**	>1024
***Klebsiella pneumoniae* ATCC 700603**	>1024

2. Chemistry supplementary results

2.1. Characterisation of scleric acid and *L*-proline-oxyacetic acid intermediate

a

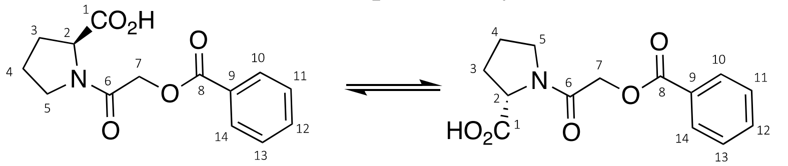

b

NMR assignment in MeOD, 700MHz, for the major scleric acid rotamer

Position, C	^{1}H (ppm, J Hz)	^{13}C (ppm)	Key HMBC
1		175.2	
2	4.49, (dd, 3.3, 8.8)	60.2	C1, C4, C6
3	2.05, 2.29 (m)	29.7	C1, C5
4	2.09, (m)	25.5	C2, C3, C5
5	3.65, 3.71 (m, m)	46.9	C2, C3, C6
6		167.6	
7	4.96, 5.10, (d, d, 15, 15)	62.9	C6, C8
8		167.1	
9		130.6	
10	8.08 (d, 8.0)	130.4	C8, C11, C13
11	7.50 (t, 8.0)	129.3	C8, C12, C14
12	7.63 (t, 8.0)	134.2	C10, C14
13	7.50 (t, 8.0)	129.3	C8, C12, C14
14	8.08 (t, 8.0)	130.4	C8, C11, C13

c

NMR assignment in MeOD, 700MHz, for the minor scleric acid rotamer

Position, C	^{1}H (ppm, J Hz)	^{13}C (ppm)	Key HMBC
1		174.6	
2	4.69, (d, 8.0)	60.1	C1, C4, C6
3	2.30, 2.37 (m, m)	32.1	C1, C5
4	1.89, 1.95, (m, m)	22.7	C2, C3, C5
5	3.57, 3.62 (m, m)	47.7	C2, C3, C6
6		167.8	
7	4.78, 5.03, (d, d, 15, 15)	62.7	C6, C8
8		167.1	
9		130.6	
10	8.08 (d, 8.0)	130.4	C8, C11, C13
11	7.50 (t, 8.0)	129.3	C8, C12, C14
12	7.63 (t, 8.0)	134.2	C10, C14
13	7.50 (t, 8.0)	129.3	C8, C12, C14
14	8.08 (t, 8.0)	130.4	C8, C11, C13

Figure S5. NMR assignment (700 MHz, CD_3OD) of scleric acid (m/z 278.1020, $C_{14}H_{15}NO_5$). (**a**) Chemical structure of scleric acid. (**b**) NMR assignment of the major rotamer. (**c**) NMR assignment of the minor rotamer of scleric acid (chemical shifts shown in light grey are those that differ from the major rotamer).

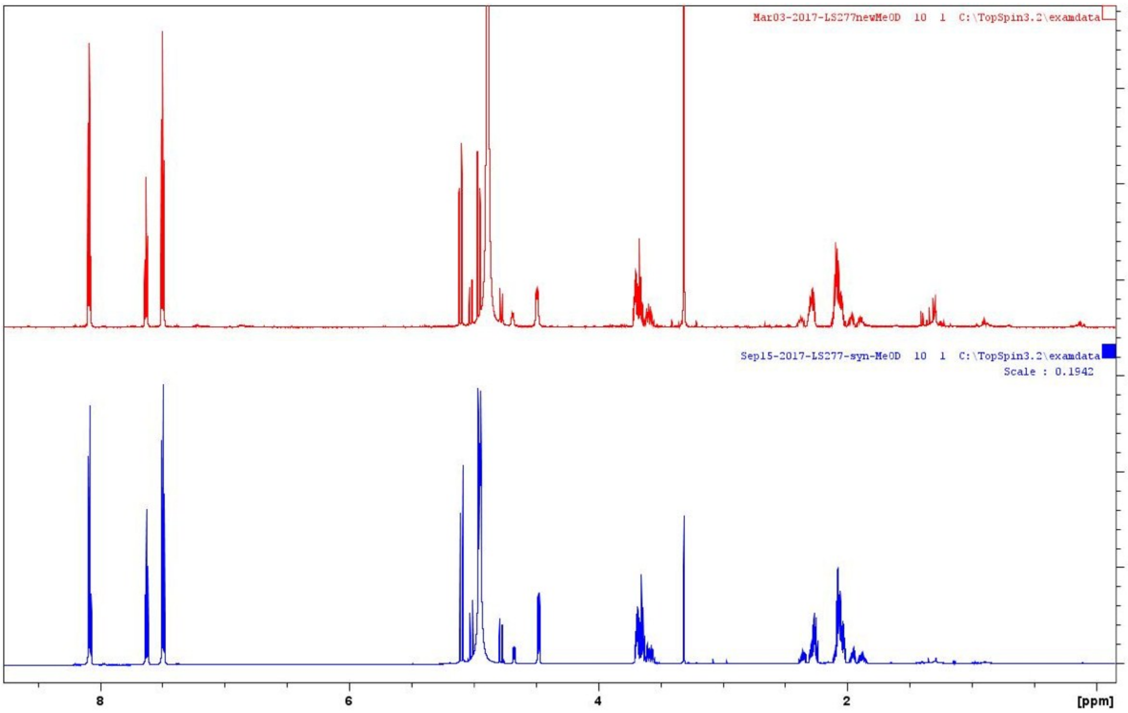

Figure S6. ^{1}H-NMR spectrum (700 MHz, CD_3OD) of scleric acid isolated from *S. albus/scl* Δ*sclM4* (top panel) and synthetic scleric acid (lower panel).

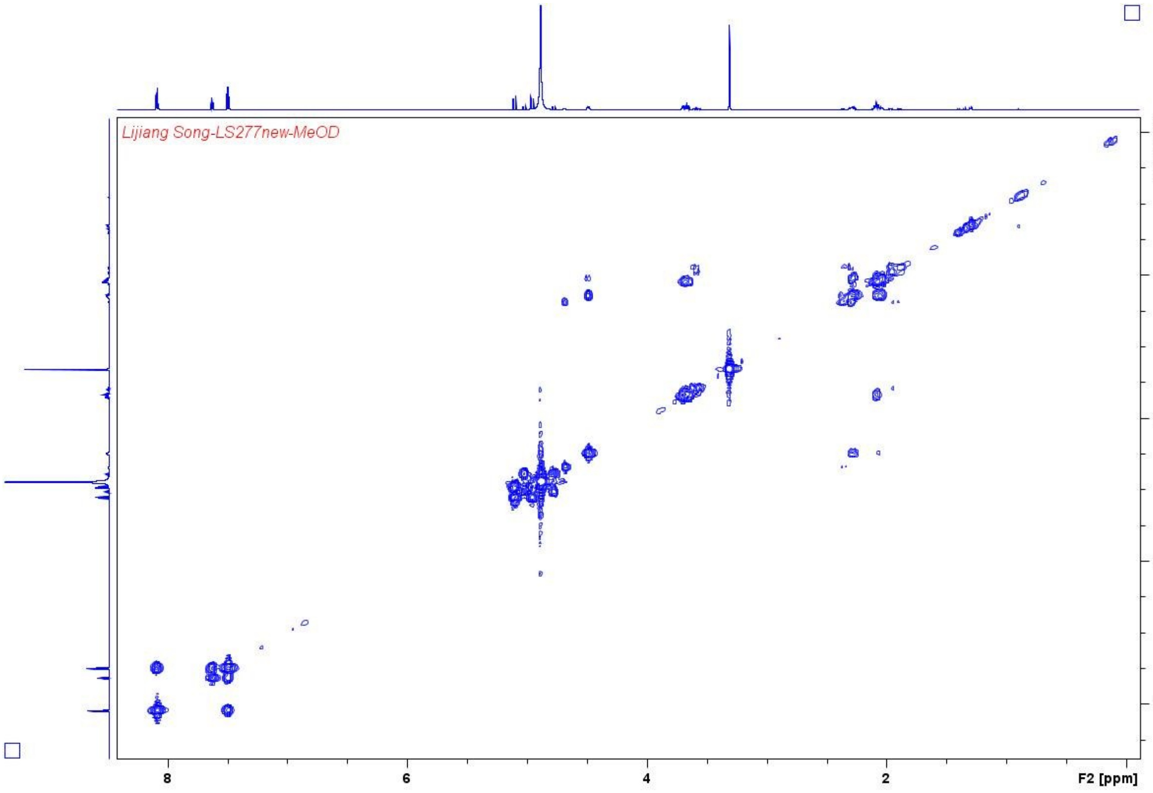

Figure S7. COSY spectrum (700 MHz, CD_3OD) of scleric acid.

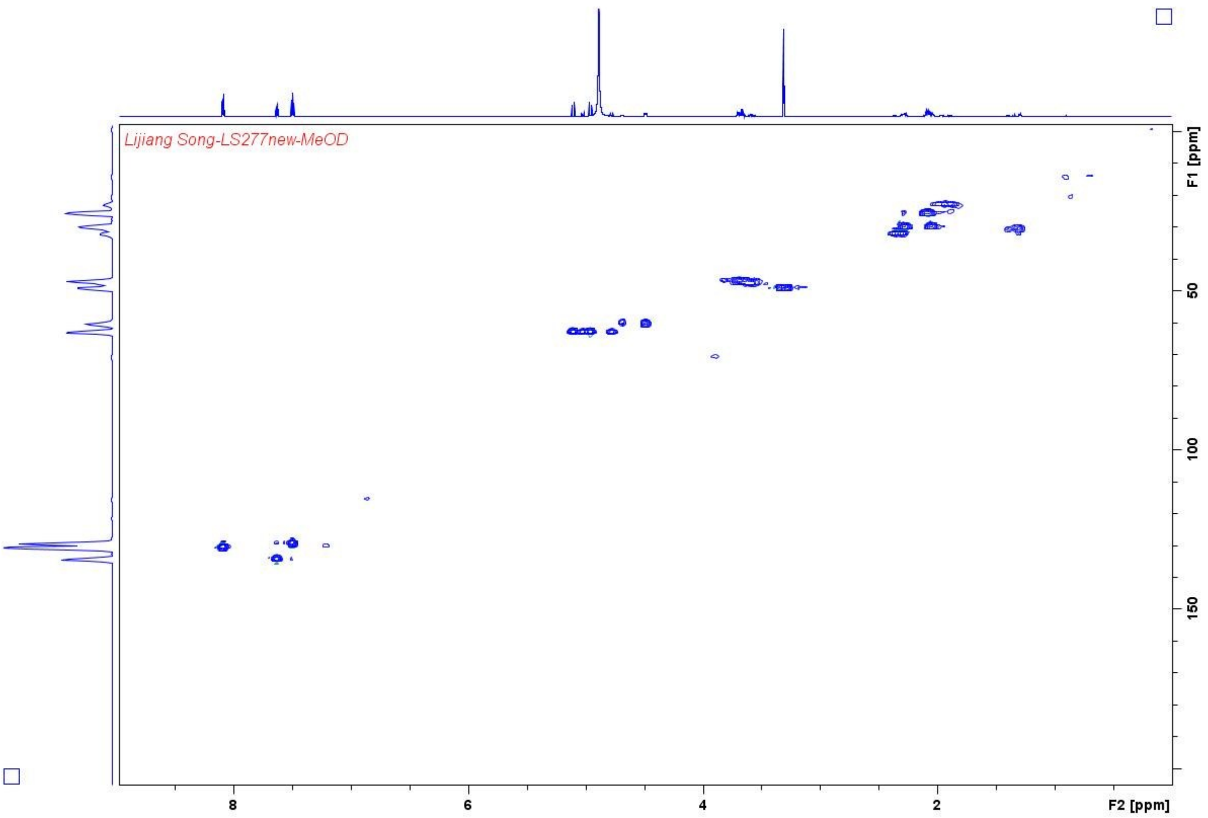

Figure S8. HSQC spectrum (700 MHz, CD_3OD) of scleric acid.

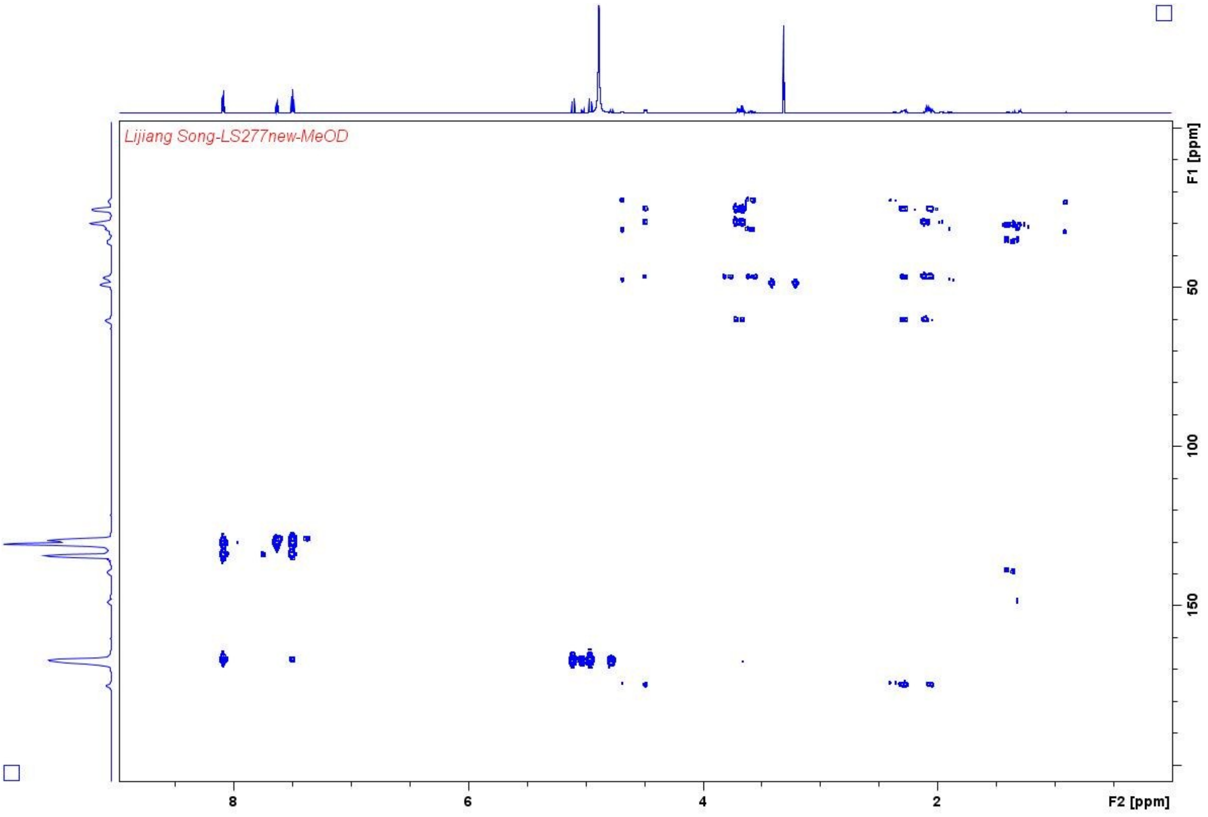

Figure S9. HMBC spectrum (700 MHz, CD_3OD) of scleric acid.

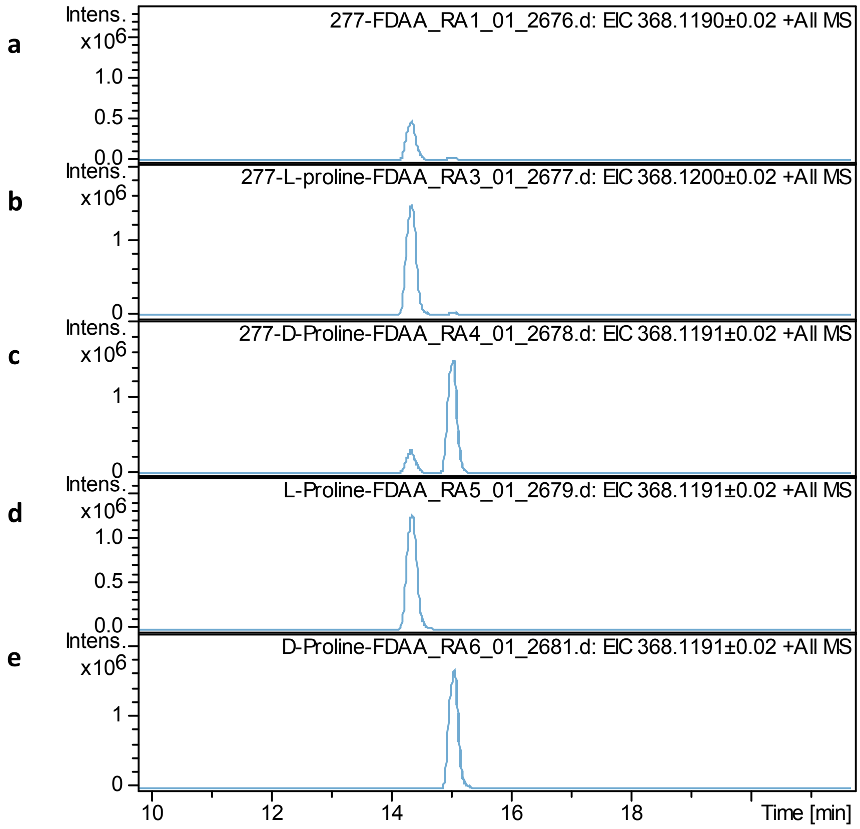

Figure S10. LC-MS analyses of proline residues derivatised with Marfey's reagent for absolute stereochemistry determination.[9]

Extracted ion chromatogram for derivatised proline originating from: (**a**) scleric acid (**b**) scleric acid and co-injected with a derivatised *L*-proline authentic standard (**c**) scleric acid and co-injected with a derivatised *D*-proline authentic standard (**d**) *L*-proline authentic standard (**e**) *D*-proline authentic standard.

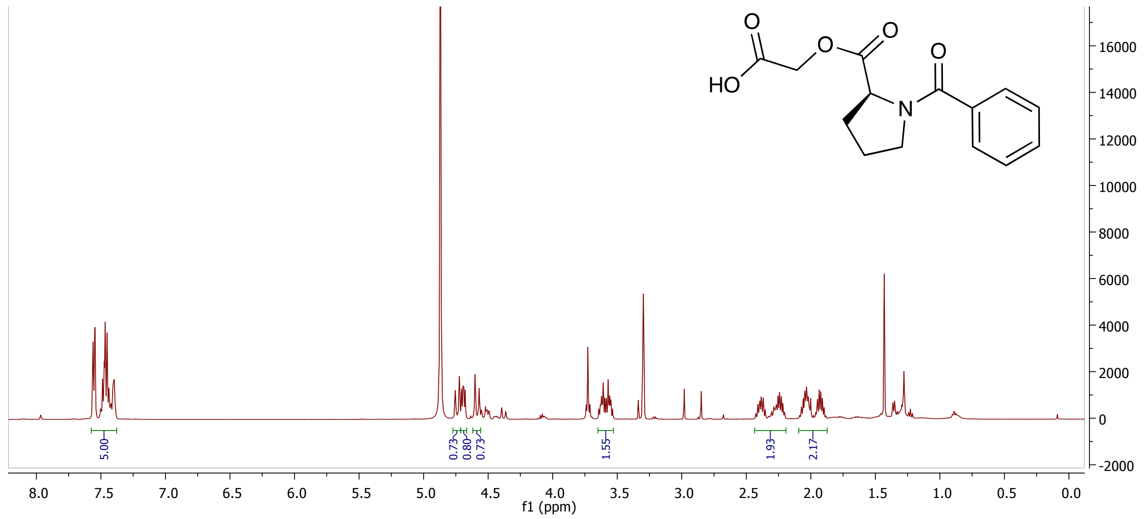

Figure S11. ^{1}H-NMR spectrum (500 MHz, CD_3OD) of the synthetic scleric acid analogue 2-((benzoyl-*L*-prolyl)oxy)acetic acid.

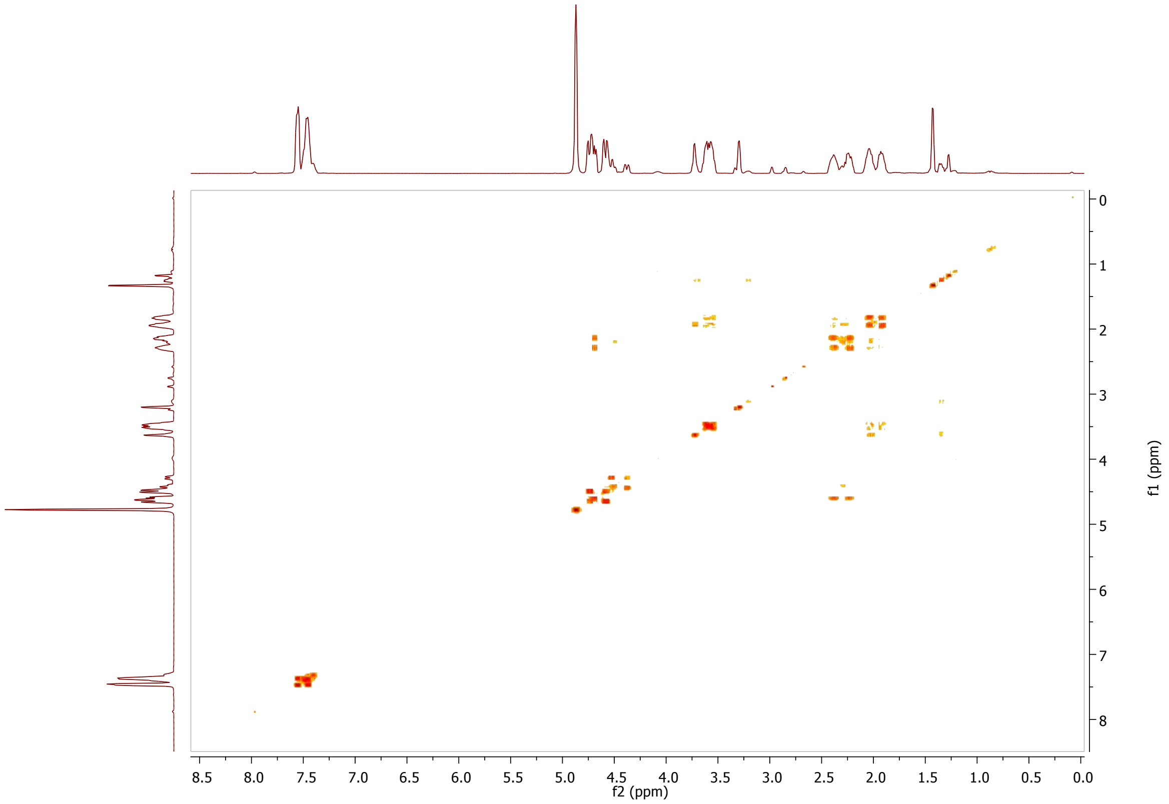

Figure S12. COSY spectrum (500 MHz, CD_3OD) of the synthetic scleric acid analogue 2-((benzoyl-*L*-prolyl)oxy)acetic acid.

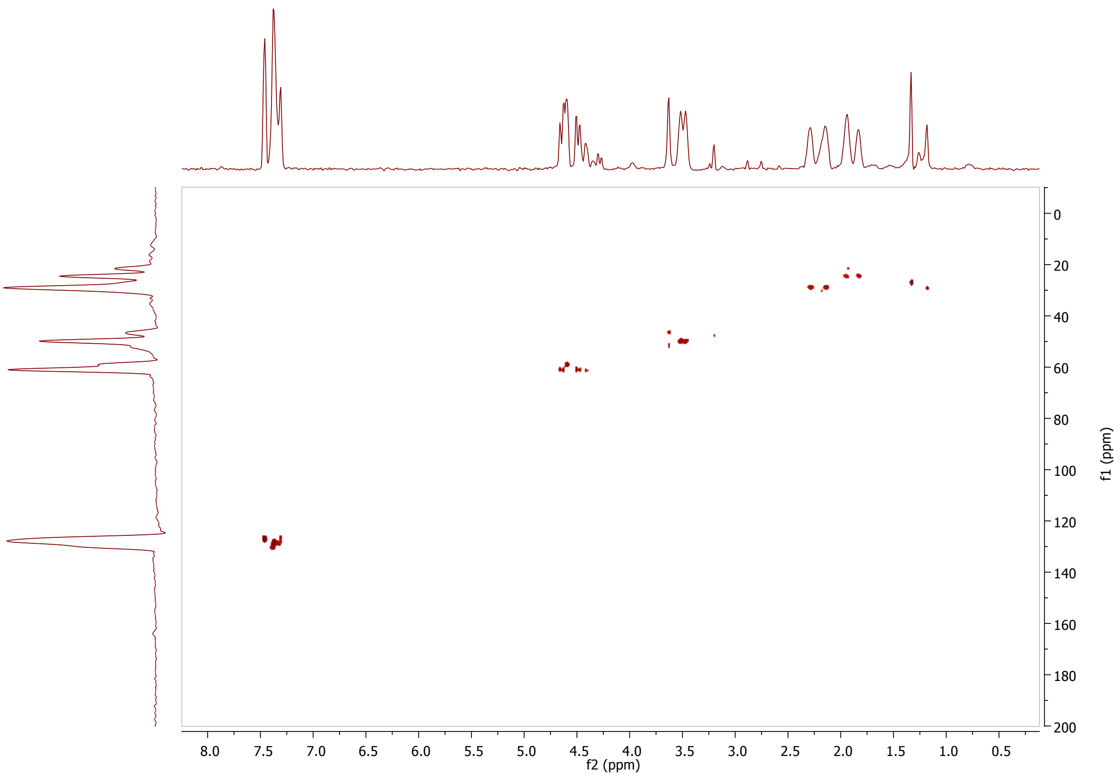

Figure S13. HSQC spectrum (500 MHz, CD_3OD) of the synthetic scleric acid analogue 2-((benzoyl-*L*-prolyl)oxy)acetic acid.

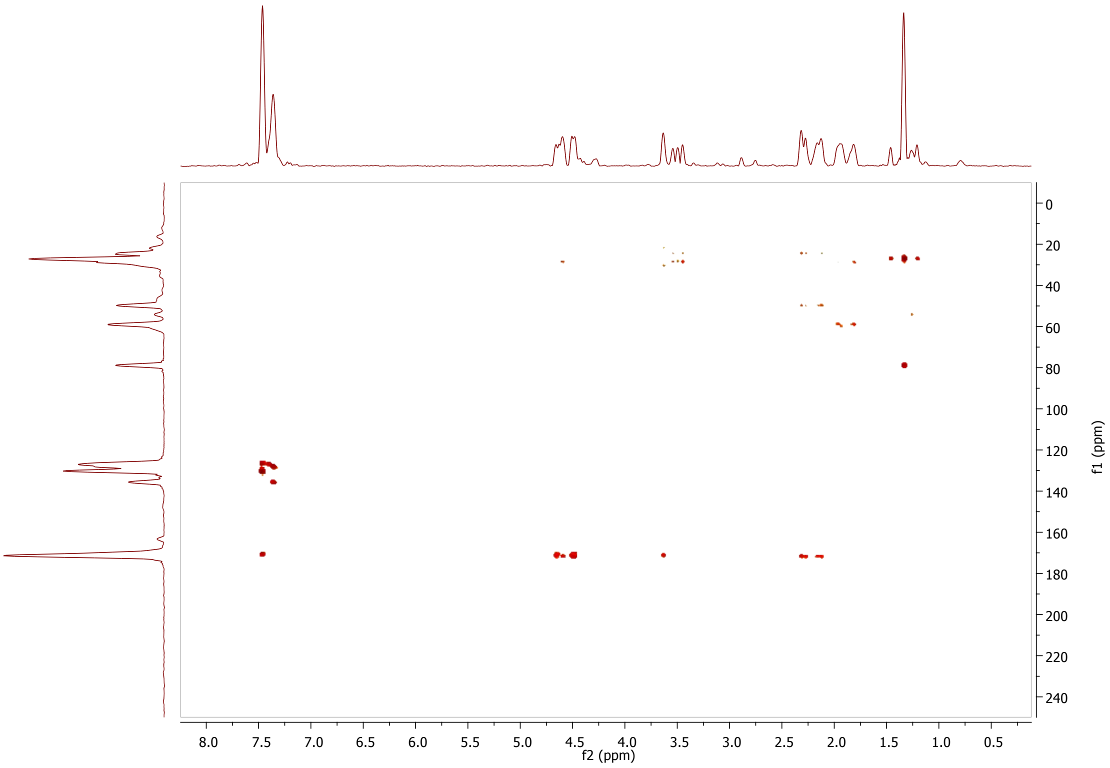

Figure S14. HMBC spectrum (500 MHz, CD_3OD) of the synthetic scleric acid analogue 2-((benzoyl-*L*-prolyl)oxy)acetic acid.

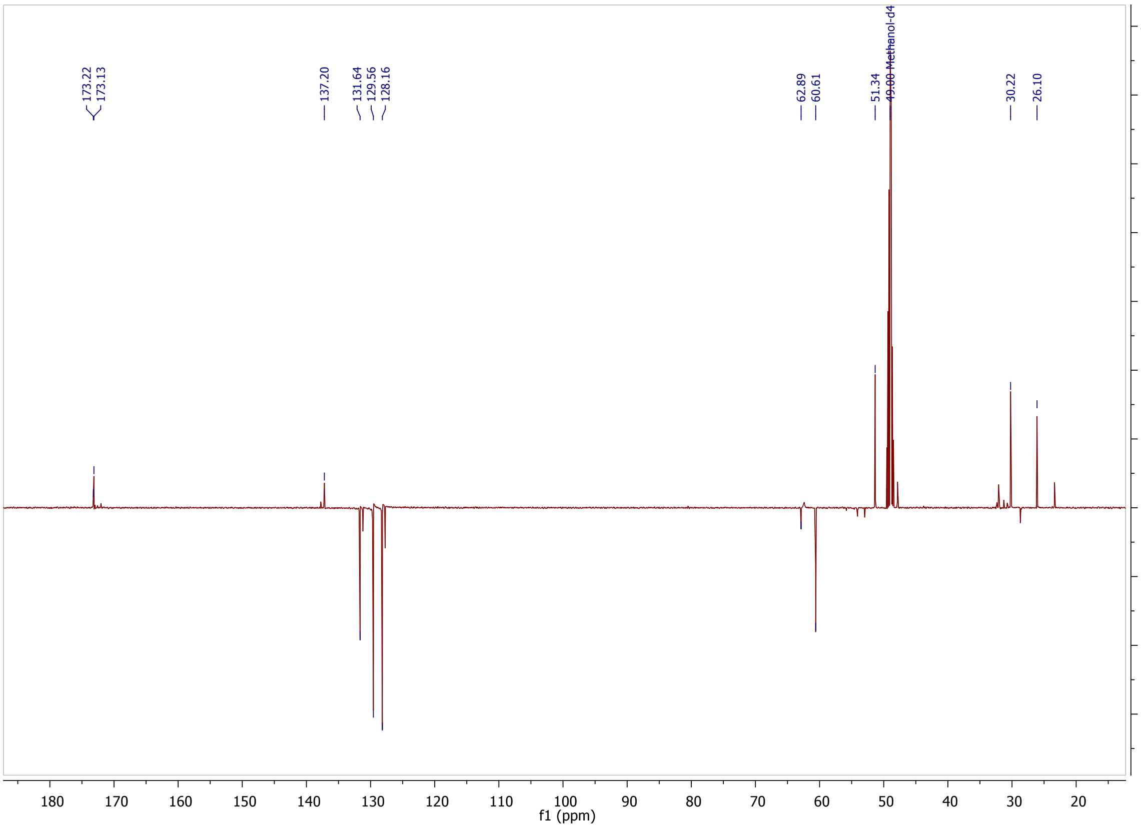

Figure S15. ^{13}C spectrum (125 MHz, CD_3OD) of the synthetic scleric acid analogue 2-((benzoyl-*L*-prolyl)oxy)acetic acid.

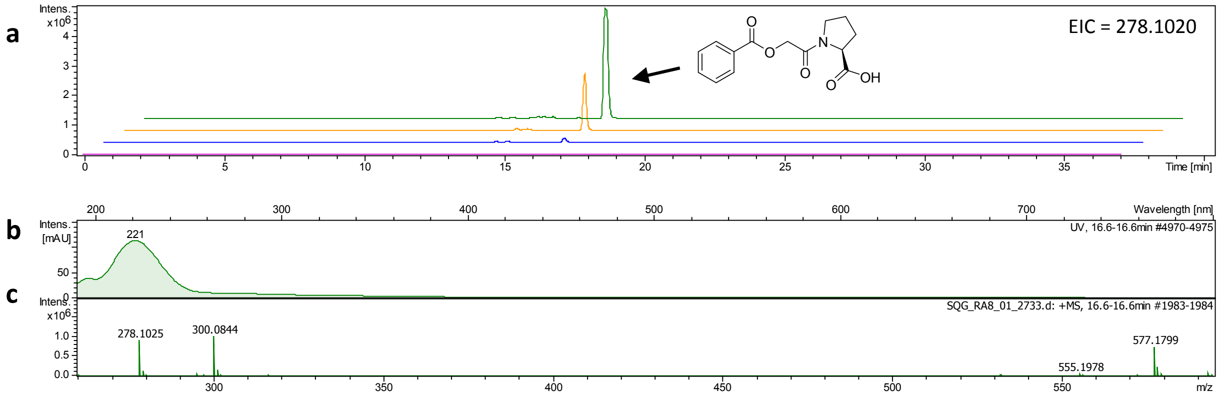

Figure S16. Restored production of scleric acid in *S. albus/scl* Δ*sclM4* Δ*Q1-4* strain upon feeding with glycolic acid.

(**a**) UHPLC-HRMS extracted ion chromatograms in positive mode for *m/z*= 278.1020 of glycolic acid standard (trace in purple), extract of *S. albus/scl* Δ*sclM4* Δ*Q1-4* grown on SM medium with no supplements (trace in blue), with 5 mM glycolic acid (trace in green) and extract of *S. albus/scl* Δ*sclM4* (trace in orange). (**b**) UV chromatogram of scleric acid. (**c**) High-resolution mass spectrometry in positive mode of scleric acid.

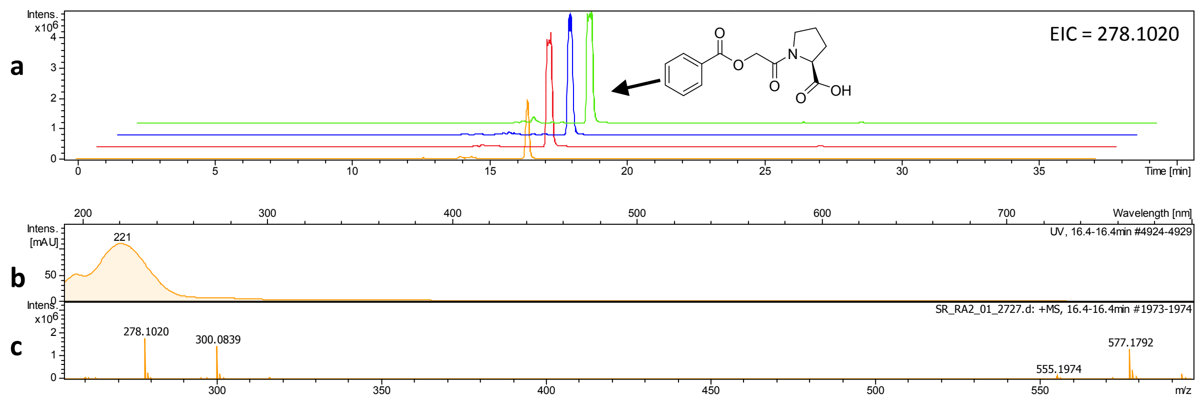

Figure S17. Scleric acid increment in precursor enriched medium detected with UHPLC-HRMS. (**a**) Extracted ion chromatograms in positive mode for *m/z*= 278.1020 of crude extracts of *S. albus/scl* Δ*sclM4* grown on SM medium with no supplements (trace in orange), with 5 mM benzoic acid (trace in red), with 5 mM glycolic acid (trace in blue), with 5 mM *L*-proline (trace in green). (**b**) UV chromatogram of scleric acid. (**c**) High-resolution mass spectrometry in positive mode of scleric acid.

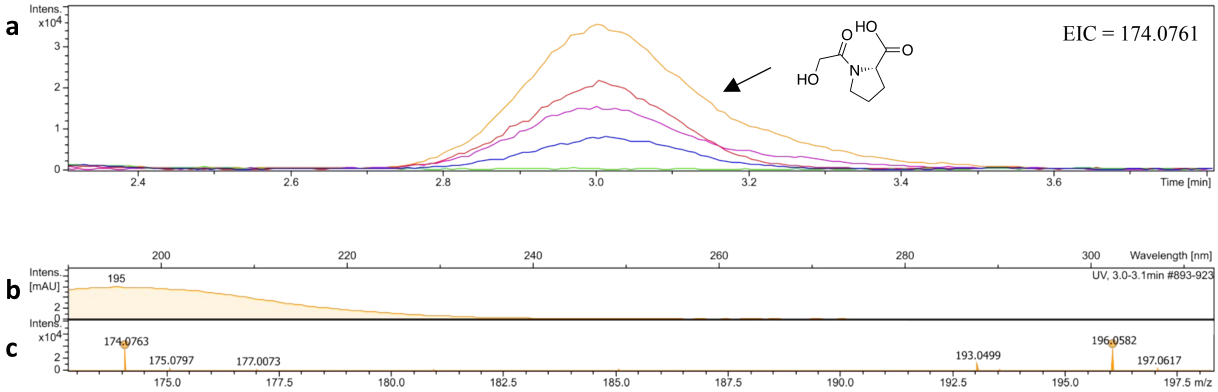

Figure S18. Detection of the *L*-proline-oxyacetic acid intermediate with UHPLC-HRMS. (**a**) Extracted ion chromatograms in positive mode for *m/z*= 174.0761 of crude extracts of *S. albus/scl* Δ*sclM4* (trace in orange), *S. albus/scl* Δ*sclM4* Δ*sclQ1-4* (blue), *S. albus/scl* Δ*sclM4* Δ*sclN* (green), *S. albus/scl* Δ*sclM4* Δ*sclA* (red) and *L*-proline-oxyacetic acid synthetic standard (purple). (**c**) UV chromatogram of *L*-proline-oxyacetic acid. (**d**) High-resolution mass spectrometry in positive mode of *L*-proline-oxyacetic acid.

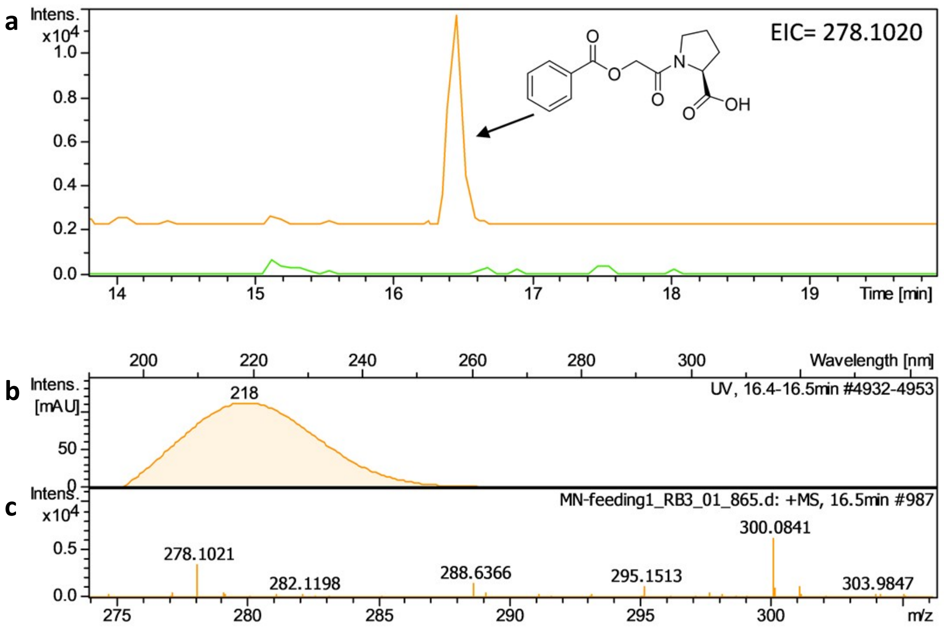

Figure S19. Detection of scleric acid by UHPLC-HRMS in *S. albus/scl* Δ*sclM4* Δ*sclN* fed with *L*-proline-oxyacetic acid.
(**a**) Extracted ion chromatograms in positive mode for *m/z*= 278.1020 of crude extracts of *S. albus/scl* Δ*sclM4* Δ*sclN* fed with 5 mM *L*-proline-oxyacetic acid (trace in orange) and control strain *S. albus/scl* Δ*sclM4* Δ*sclN* (trace in green). (**b**) UV chromatogram of scleric acid. (**c**) High-resolution mass spectrometry in positive mode of scleric acid.

2.2. Synthetic Chemistry

All chemicals were purchased from Sigma-Aldrich, VWR, Alfa Aesar, Fluorochem or Carbosynth and used without further purification. Dry solvents were purchased from Fisher Scientific or dried using solvent towers. Reagent grade solvents were purchased from Fisher Scientific.
Analytical TLC was performed on aluminium sheets precoated with silica gel 60 (F_{254}, Merck) and visualised under UV light (short wave) and using potassium permanganate or ninhydrin stains. Silica gel was purchased from Sigma-Aldrich (Tech grade, pore size 60 Å, 230-400 mesh).
^{1}H, ^{13}C and ^{19}F NMR spectra were recorded in d_4-MeOD or $CDCl_3$ on the following Bruker Avance instruments: DPX-300, DPX-400, DRX-500 or AV-600.
High-resolution mass spectra (HRMS) were obtained using electrospray ionisation (ESI) on a MaXis UHR-TOF (Bruker Daltonics) or on a Bruker MaXis (ESI-HR-MS).
Optical rotations were obtained using an AA-1000 Polarimeter from Optical Activity Ltd.

2.2.1. Synthesis of scleric acid

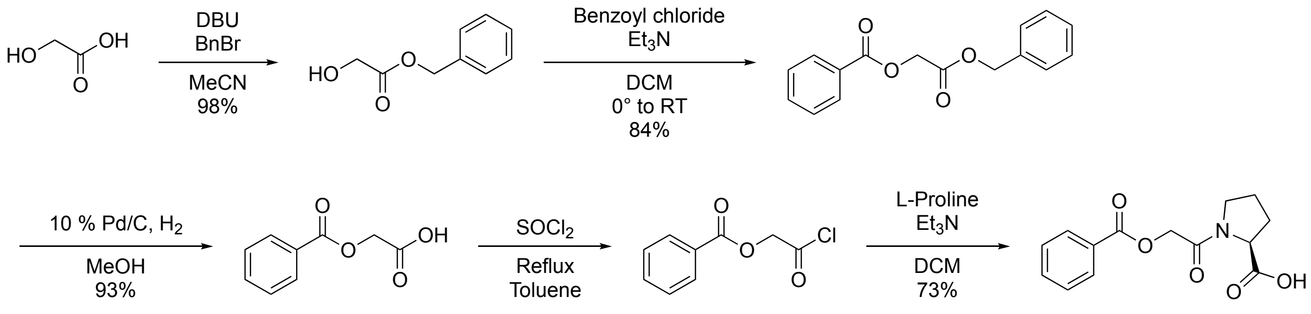

Figure S20. Schematic representation of synthetic route to scleric acid.

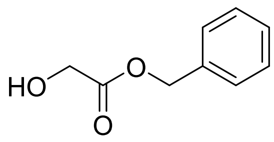

Benzyl 2-hydroxyacetate

Glycolic acid (1.000 g, 13.15 mmol) under Argon was dissolved in acetonitrile, and benzyl bromide (1.25 mL, 10.51 mmol) was added and cooled to 0 °C. 1,8-Diazabicyclo(5.4.0)undec-7-ene (DBU, 1.57 mL, 10.51 mmol) was then added dropwise and allowed to return to room temperature over 2 hours. The solvent was removed *in vacuo* and the residue redissolved in EtOAc (40 mL) and H_2O (10 mL). The layers were separated, and the organic layer was washed with 1M HCl (40 mL) and brine (2 x 40 mL), dried on $MgSO_{4(s)}$, and finally concentrated to afford benzyl 2-hydroxyacetate as a colourless oil (1.711 g, 98 %). The characterisation data were in accordance with those previously reported in the literature.[10]

^{1}H NMR (300 MHz, $CDCl_3$): δ 7.41 – 7.32 (5H, s, Ar*H*), 5.24 (2H, s, C*H*$_2$), 4.20 (2H, d, 5.4 Hz, C*H*$_2$OH), 2.34 (1H, t, 5.4 Hz, CH_2O*H*); **LRMS (ESI):** calculated for $C_9H_{10}O_3Na$: 189.1, found: 188.7.

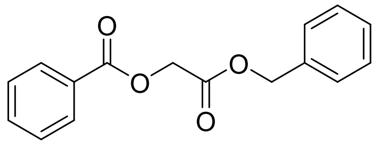

2-(benzyloxy)-2-oxoethyl benzoate

Benzyl 2-hydroxyacetate (1.711 g, 10.30 mmol) was dissolved in anhydrous dichloromethane (DCM, 30 mL) under an Argon atmosphere and cooled to 0 °C. Triethylamine (Et_3N, 1.66 mL, 11.90 mmol) was added, and benzoyl chloride (1.32 mL, 11.30 mmol) was added dropwise. The mixture was allowed to return to room temperature and stirred for 5 hours. 1M HCl (30 mL) was added to the reaction mixture, and the layers were separated. The organic phase was washed with brine (30 mL), dried on $MgSO_{4(s)}$, filtered and concentrated to afford 2-(benzyloxy)-2-oxoethyl benzoate as a white solid (2.452 g, 84 %).

The characterisation data were in accordance with those previously reported in the literature.[11]

^{1}H NMR (300 MHz, $CDCl_3$): δ 8.09 (2H, m, Ar*H*), 7.59 (1H, m, Ar*H*), 7.46 (2H, m, Ar*H*), 7.36 (5H, m, Ar*H*), 5.24 (2H, s, C*H*$_2$OCO), 4.90 (2H, s, COC*H*$_2$O); **LRMS (ESI):** calculated for $C_{16}H_{14}O_4Na$ $[M+Na]^+$: 293.1, found: 292.8.

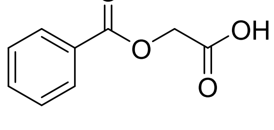

2-(benzoyloxy)acetic acid

2-(benzyloxy)-2-oxoethyl benzoate (992 mg, 3.67 mmol) and 10 % Pd/C (312 mg, 2.94 mmol) under an Argon atmosphere were dissolved in anhydrous MeOH, and Argon gas bubbled through the solution for 10 minutes. The argon atmosphere was then replaced with a hydrogen atmosphere and the reaction stirred at room temperature overnight. The mixture was then filtered through Celite, the Celite pad

washed with MeOH and the filtrate concentrated to afforded 2-(benzoyloxy)acetic acid as a white solid (612 mg, 93 %).

The characterisation data were in accordance with those previously reported in the literature.[12]

^{1}H NMR (300 MHz, MeOD): δ 8.06 (2H, m, Ar*H*), 7.57 (1H, m, Ar*H*), 7.43 (2H, m, Ar*H*), 4.87 (2H, s, COCH_2O); **LRMS (ESI):** calculated for $C_9H_7O_4$ [M-H]$^-$: 179.0, found: 179.0.

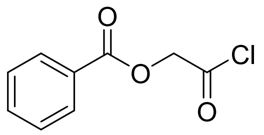

2-chloro-2-oxoethyl benzoate

2-(benzoyloxy)acetic acid (251 mg, 1.39 mmol) was dissolved in anhydrous toluene (10 mL) under an Argon atmosphere, and thionyl chloride (1 ml, 5.15 mmol) was added. The mixture was heated to reflux for 3 hours and then concentrated to dryness to afford crude 2-chloro-2-oxoethyl benzoate as a brown oil. The material was used directly in the next step without further purification.

The characterisation data were in accordance with those previously reported in the literature.[12]

^{1}H NMR (300 MHz, $CDCl_3$): δ 8.07 (2H, m, Ar*H*), 7.63 (1H, m, Ar*H*), 7.48 (2H, m, Ar*H*), 5.15 (OCH_2COCl).

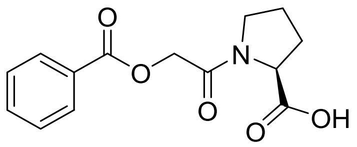

Scleric acid ((2-(benzoyloxy)acetyl)-*L*-proline)

L-proline (151 mg, 1.32 mmol) was suspended in anhydrous DCM (10 ml) under Argon. Et_3N was added dropwise, and the mixture cooled to 0 °C. 2-chloro-2-oxoethyl benzoate (276 mg, 1.39 mmol) was dissolved in anhydrous DCM (6 ml) and added dropwise to the proline suspension and stirred for 3 hours. This organic layer was washed with 1M HCl (15 ml) and brine (15 ml), dried on $MgSO_{4(s)}$, filtered and concentrated. The residue was purified by silica gel chromatography eluting with EtOAc to afford scleric acid as a white solid (269 mg, 73 %). Rf: 0.23 in 1:9 MeOH:DCM; $[\alpha]_D^{20}$ = -65 (c 0.195, MeOH);

Major rotamer: **^{1}H NMR** (700 MHz, MeOD): δ 8.09 (2H, m, COCC*H*), 7.63 (1H, m, COCCHCHC*H*), 7.50 (2H, m, COCCHC*H*), 5.10 (1H, dd, 15.0 Hz, COCH_2O), 4.95 (1H, dd, 15.0 Hz, COCH_2O), 4.48 (1H, dd, 9 Hz, 3.4 Hz, NC*H*), 3.70 – 3.63 (2H, m, NCH_2), 2.31 – 2.24 (1H, m, NCHCH_2), 2.10 – 2.01 (3H, m, NCHCH_2, NCH$_2$CH_2); **^{13}C NMR** (125 MHz, MeOD): 175.4 (*C*O$_2$H), 168.1 (N*C*O), 167.5 (OCOC(CH)$_2$), 134.5 (COCCHCHC*H*), 130.8 (COC*C*H), 130.8 (OCO*C*CH), 129.6 (COCCH*C*H), 63.1 (CO*C*H$_2$O), 60.5 (N*C*H), 47.1 (N*C*H$_2$), 30.0 (NCH*C*H$_2$), 25.7 (NCH$_2$*C*H$_2$); Minor rotamer: **^{1}H NMR** (700 MHz, MeOD): δ 8.09 (2H, m, COCC*H*), 7.63 (1H, m, COCCHCHC*H*), 7.50 (2H, m, COCCHC*H*), 5.04 (1H, dd, 14.7 Hz, COCH_2O), 4.80 (1H, dd, 14.7 Hz, COCH_2O), 4.69 (1H, dd, 2.3 Hz, 8.5 Hz, NC*H*), 3.60 (2H, m, NCH_2), 2.37 (1H, m, NCHCH_2), 1.93 (3H, m, NCHCH_2, NCH$_2$CH_2); **^{13}C NMR** (125 MHz, MeOD): 174.8 (*C*O$_2$H), 168.6 (N*C*O), 167.5 (O*C*OC(CH)$_2$), 134.5 (COCCHCH*C*H), 130.8 (COC*C*H), 130.8 (OCO*C*CH), 129.6 (COCCH*C*H), 63.3 (CO*C*H$_2$O), 60.1

(N*C*H), 48.0 (N*C*H_2), 32.3 (NCH*C*H_2), 23.1 (NCH_2*C*H_2); **HRMS (ESI)**: calculated for $C_{14}H_{15}O_5NNa$ $[M+Na]^+$: 300.0842, found: 300.0842.

2.2.2. Synthesis of the scleric acid structural isomer

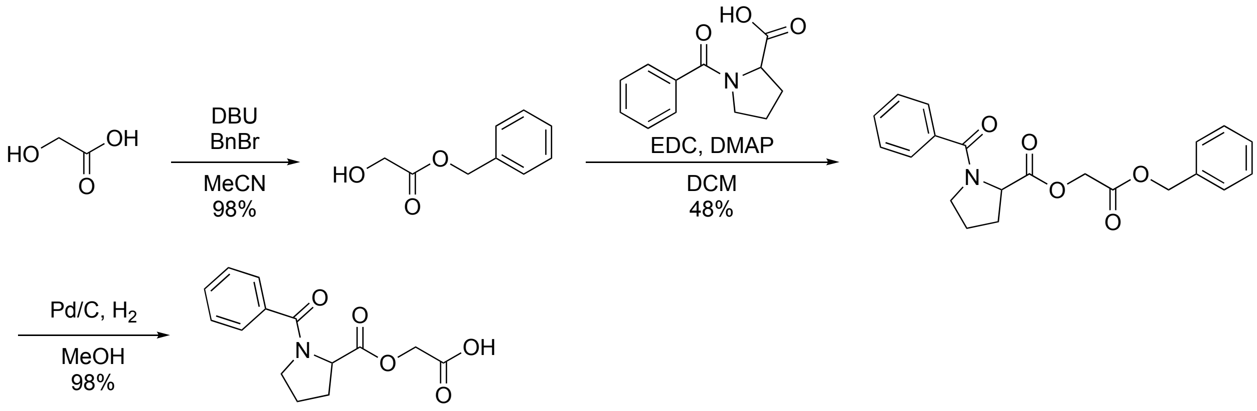

Figure S21. Schematic representation of synthetic route to the scleric acid structural isomer 2-((benzoyl-prolyl)oxy)acetic acid.

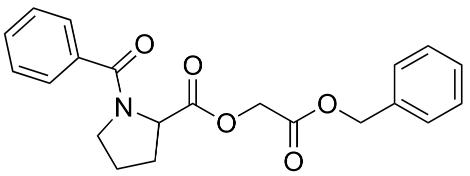

2-(benzyloxy)-2-oxoethyl benzoylprolinate

Benzyl 2-hydroxyacetate (18 mg, 0.109 mmol), 1-benzoyl-pyrroldine-2-carboxylic acid (20 mg, 0.091 mmol) and 4-dimethylaminopyridine (DMAP, 2.2 mg, 0.018 mmol) under an Argon atmosphere were dissolved in anhydrous DCM (1 mL). 1-Ethyl-3-(3-dimethylaminopropyl)carbodiimide (EDC) (21 µL, 0.119 mmol) was added, and the mixture stirred at room temperature overnight. The mixture was then diluted with DCM and washed with sat. $NaHCO_3$ (5 mL), 0.1 M HCl (5 mL) and brine (5 mL). The organic layer was dried on $MgSO_{4(s)}$, filtered and concentrated. The residue was purified using silica gel chromatography (eluting from 3:1 Petroleum ether:EtOAc to 3:2 Petroleum ether:EtOAc) to afford 2-(benzyloxy)-2-oxoethyl benzoylprolinate as a white solid (16 mg, 49 %).

^{1}H NMR (500 MHz,): δ 7.57-7.38 (5H, m, Ar*H*), 4.74 (1H, dd, 15.8 Hz, COC*H_2*O), 4.69 (1H, 8.5 Hz, 5.2 Hz, NC*H*CO), 4.58 (1H, dd, 15.8 Hz, COC*H_2*O), 3.65-3.53 (2H, m, NC*H_2*), 2.43-2.10 (2H, m, NCHC*H_2*), 2.09-1.79 (2H, m, NCH_2C*H_2*); **^{13}C NMR** (125 MHz,): δ 173.2 (*C*OOH), 173.2 (CH*C*OO), 137.2 (CH_2*C*CHCH), 131.6 (Ar), 129.6 (Ar), 128.1 (Ar), 62.9 (*C*H_2COOH), 60.6 (N*C*H), 51.3 (N*C*H_2), 30.2 (NCH*C*H_2), 26.1 (NCH_2*C*H_2); **HRMS(ESI):** calculated for $C_{21}H_{21}NO_5Na$ $[M+Na]^+$: 390.1312, found: 390.1312.

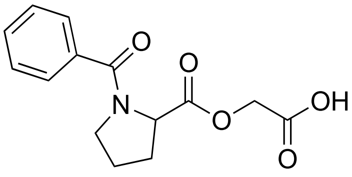

2-((benzoylprolyl)oxy)acetic acid

2-(benzyloxy)-2-oxoethyl benzoylprolinate (16 mg, 0.058 mmol), 10% Pd/C (5 mg, 0.046 mmol) under an Argon atmosphere were dissolved in dry MeOH (2 mL), and the atmosphere replaced with hydrogen. The mixture was stirred at room temperature overnight, then filtered through Celite and concentrated to afford 2-((benzoylprolyl)oxy)acetic acid as a colourless oil (12 mg, 98 %).

^{1}H NMR (500 MHz, MeOD): δ 7.57 -7.38 (5H, m, Ar*H*), 4.74 (1H, dd, 15.8 Hz, C*H*$_2$COOH), 4.58 (1H, dd, 15.8 Hz, C*H*$_2$COOH), 4.69 (1H, dd, 5.2 Hz, 8.5 Hz, NC*H*COO), 3.65 – 3.53 (2H, m, C*H*$_2$N), 3.43 – 2.19 (2H, m, C*H*$_2$CHN), 2.09 – 1.79 (2H, m, C*H*$_2$CH$_2$N); **^{13}C NMR** (125 MHz, MeOD) δ 173.2 (CH$_2$*C*OOH), 173.2 (N*C*HCOO), 173.1 (Ar*C*ON), 137.2 (CHCH*C*CO), 131.6 (*C*HCCO), 129.6 (*C*HCHCHCCO), 128.1 (*C*HCHCCO), 62.9 (*C*H$_2$COOH), 60.6 (N*C*HCOO), 46.9 (*C*H$_2$N), 29.7 (*C*H$_2$CHN), 25.5 (*C*H$_2$CH$_2$N); **HRMS(ESI)**: calculated for $C_{14}H_{15}NO_5Na$ [M+Na]$^+$: 300.0842, found: 300.0843.

2.2.3.Synthesis of the *L*-proline-oxyacetic acid intermediate

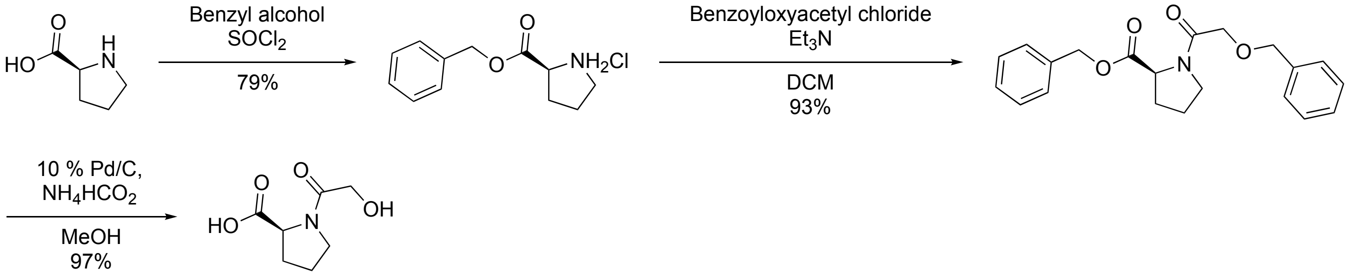

Figure S22. Schematic representation of synthetic route to *L*-proline-oxyacetic acid.

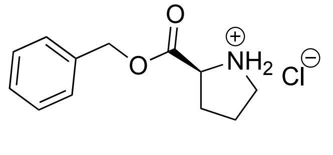

Benzyl prolinate hydrochloride

Benzyl alcohol (13.5 mL, 130.4 mmol) was cooled to 0 °C and SOCl$_2$ (1.27 mL, 17.4 mmol) was added dropwise. *L*-proline (1.00 g, 8.69 mmol) was added in one portion and the mixture stirred at 0 °C for 2 hours, then for further 24 hours at room temperature. The mixture was cooled to -20 °C and the product was triturated in Et$_2$O (150 mL) to afford benzyl prolinate hydrochloride as a white solid (1.653 g, 79%).

The characterisation data were in accordance with those previously reported in the literature.[13]

^{1}H NMR (300 MHz, MeOD): δ 7.40 (5H, m, Ar*H*), 5.29 (2H, dd, 12.1 Hz, 4.0 Hz, ArC*H*$_2$O), 4.49 (1H, t, 7.7 Hz, OOCC*H*NH), 3.39 (2H, m, NC*H*$_2$), 2.44 (1H, m, NHCHC*H*$_2$), 2.09 (3H, m, NHCHC*H*$_2$, NHC*H*$_2$); **LRMS (ESI):** calculated for $C_{12}H_{16}O_2N$ [M+H]$^+$: 206.1, found: 205.6.

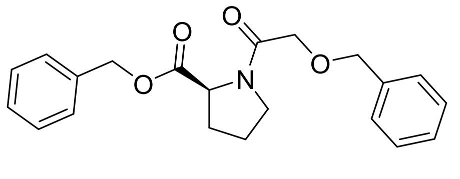

benzyl (2-(benzyloxy)acetyl)-*L*-prolinate

Benzyl prolinate hydrochloride (500 mg, 2.07 mmol) was suspended in DCM under an inert atmosphere, and Et$_3$N (606 µL, 4.35 mmol) was added. The mixture was cooled to 0 °C, and benzyloxyacetyl chloride (402 µL, 2.18 mmol) was added dropwise. The reaction mixture was allowed

to warm to room temperature and then stirred overnight. The reaction mixture was concentrated and the crude residue redissolved in EtOAc (25 mL). The organic phase was washed with 1M HCl (20 mL), brine (20 mL) and sat. $NaHCO_3$ (20 mL), and then dried on $MgSO_{4(s)}$, filtered and concentrated. The resulting residue was purified by silica gel chromatography (1:4 EtOAc:Petroleum ether to 1:1 EtOAc:Petroleum ether) to afford benzyl (2-(benzyloxy)acetyl)-*L*-prolinate as a colourless oil (681 mg, 93%) with a 3:1 mixture of rotamers. **R_f**: 0.30 in 1:1 EtOAc: Petroleum ether.

Major rotamer: **^{1}H NMR** (500 MHz, $CDCl_3$): δ 7.37 – 7.27 (10H, m, Ar*H*), 5.18 (2H, dd, 12.3 Hz, 8.9 Hz, C*H_2*OCO), 4.62 (2H, m, CH_2OC*H_2*Ar), 4.60 (1H, m, NHC*H*COO), 4.16 (2H, dd, 14.3 Hz, 3.2 Hz, COC*H_2*O), 3.62 (1H, m, NC*H_2*), 3.52 (1H, m, NC*H_2*), 2.17 (1H, m, NCHC*H_2*), 1.96 (1H, m, NCHC*H_2*), 2.03 (1H, m, NCH_2C*H_2*), 1.95 (1H, m, NCH_2C*H_2*); **^{13}C NMR** (125 MHz, $CDCl_3$): δ 172.1 (CH*C*OO), 168.5 (N*C*OCH_2), 137.5 (CH_2OCH_2*C*$(CH)_2$), 135.8 (COOCH_2*C*$(CH)_2$), 128.8 (CH), 128.7 (CH), 128.6 (CH), 128.6 (CH), 128.5 (CH), 128.4 (CH), 128.3 (CH), 128.2 (CH), 128.1 (CH), 128.0 (CH), 73.2 (CH_2O*C*H_2C$(CH)_2$), 69.6 (NCO*C*H_2), 67.0 (COO*C*H_2C$(CH)_2$), 59.2 (N*C*H), 46.5 (N*C*H_2), 28.9 (NCH*C*H_2), 25.1 (NCH_2*C*H_2);
Minor rotamer: **^{1}H NMR** (500 MHz, $CDCl_3$): δ 7.37 – 7.27 (10H, m, Ar*H*), 5.04 (1H, dd, 12.1 Hz, C*H_2*OCO), 4.92 (1H, dd, 12.2 Hz, C*H_2*OCO), 4.60 (1H, m, NHC*H*COO), 4.42 (2H, m, CH_2OC*H_2*Ar), 4.04 (2H, s, COC*H_2*O), 3.68, 3.62 (2H, m, NC*H_2*), 2.19, 2.11 (2H, m, NCHC*H_2*), 1.86 (2H, m, NCH_2C*H_2*); **^{13}C NMR** (125 MHz, $CDCl_3$): δ 172.1 (CH*C*OO), 168.6 (N*C*OCH_2), 137.2 (CH_2OCH_2*C*$(CH)_2$), 135.5 (COOCH_2*C*$(CH)_2$), 128.8 (CH), 128.7 (CH), 128.6 (CH), 128.6 (CH), 128.5 (CH), 128.4 (CH), 128.3 (CH), 128.2 (CH), 128.1 (CH), 128.0 (CH), 73.4 (CH_2O*C*H_2C$(CH)_2$), 70.7 (NCO*C*H_2), 67.1 (COO*C*H_2C$(CH)_2$), 59.2 (N*C*H), 47.1 (N*C*H_2), 31.7 (NCH*C*H_2), 22.0 (NCH_2*C*H_2); **HRMS (ESI)**: calculated for $C_{21}H_{23}O_4NNa$ $[M+Na]^+$: 376.1519, found: 376.1522.

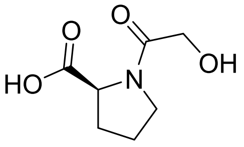

***L*-proline-oxyacetic acid intermediate (**(2-hydroxyacetyl)-*L*-proline)

Benzyl (2-(benzyloxy)acetyl)-*L*-prolinate (51 mg, 0.141 mmol), 10 % Pd/C (12 mg, 0.113 mmol) and ammonium formate (46 mg, 0.707 mmol) under an Argon atmosphere were dissolved in anhydrous MeOH (5 ml), and heated to reflux for 4 hours. The mixture was cooled to room temperature and filtered trhough Celite to afford (2-hydroxyacetyl)-*L*-proline as a hygroscopic white solid (24 mg, 97 %) and a 1:1 mixture of rotamers which were not distinguishable by NMR.

^{1}H NMR (400 MHz, MeOD): δ 4.36 (1H, dd, 3.2 Hz, 8.7 Hz, NC*H*), 4.19 (2H, dd, 15.6 Hz, 6.5 Hz, COC*H_2*OH), 4.16 (1H, dd, 3.4 Hz, 7.7 Hz, NC*H*), 4.09 (2H, dd, 15.4 Hz, 16.2 Hz, COC*H_2*OH), 3.63, 3.53 (2H, m, NC*H_2*), 3.53, 3.42 (2H, m, NC*H_2*), 2.25, 2.15 (2H, m, NCHC*H_2*), 2.15, 2.00 (2H, m, NCHC*H_2*), 2.01, 1.94 (2H, m, NCH_2C*H_2*), 1.90, 1.86 (2H, m, NCH_2C*H_2*); **^{13}C NMR** (100 MHz, MeOD): δ 179.3 (*C*OOH), 178.8 (*C*OOH), 172.9 (N*C*OCH_2), 172.1 (N*C*OCH_2), 62.8 (N*C*H), 62.2 (N*C*H), 61.8 (CO*C*H_2OH), 61.5 (CO*C*H_2OH), 47.9 (N*C*H_2), 46.6 (N*C*H_2), 33.0 (NCH*C*H_2), 30.7 (NCH*C*H_2), 25.6 (NCH_2*C*H_2), 23.4 (NCH_2*C*H_2); **HRMS (ESI)**: calculated for $C_7H_{11}O_4NNa$: 196.0580, found: 196.0583.

References

1. M.J. Thirumalachar, *Nature*, 1955, **176**, 934.
2. N. Zaburannyi, M. Rabyk, B. Ostash, V. Fedorenko and A. Luzhetskyy, *BMC Genomics* 2014, **15**, 97.
3. J.P. Gomez-Escribano, M.J. Bibb, *Microbial. Biotechnol.,* 2011, **4**, 207.
4. D.J. MacNeil, J.L. Occi, K.M. Gewain, T. MacNeil, P.H. Gibbons, C.L. Ruby, S.J. Danis, *Gene* 1992, **115**, 119.
5. F. Flett, V. Mersinias, C.P., *FEMS Microbiol. Lett.*, 1997, **155**, 223.
6. X. Tang, J. Li, N. Millán-Aguiñaga, J.J. Zhang, E.C. O'Neill, J.A. Ugalde, P.R. Jensen, S.M. Mantovani, and B. S. Moore, *ACS Chem. Biol.,* 2015, **10**, 2841.
7. R.E. Cobb, Y. Wang and H. Zhao, *ACS Synth. Biol.*, 2015, **4**, 723.
8. E.L.C. de los Santos and G.L. Challis, *bioRxiv*, 2017, doi: 10.1101/119214.
9. P. Marfey, *Carlsberg Res. Commun.*, 1984, **49**, 591.
10. M. Gynther, J. Ropponen, K. Laine, J. Leppänen, P. Haapakoski, L. Peura, T. Järvinen and J. Rautio, *J. Med. Chem.*, 2009, **52**, 3348.
11. B. J. W. Barratt, C. J. Easton, D. J. Henry, I. H. W. Li, L. Radom and J. S. Simpson, *J. Am. Chem. Soc.*, 2004, **126**, 13306.
12. S. J. Danishefsky, W. H. Pearson and B. E. Segmullert, *J. Am. Chem. Soc.*, 1985, **5**, 1280.
13. Z. Li, I. O. Lebedyeva, V. M. Golubovskaya, W. G. Cance, K. A. Alamry, H. M. Faidallah, C. Dennis Hall and A. R. Katritzky, *Bioorganic Med. Chem.*, 2015, **23**, 5056.
